# Regional and subtype-dependent miRNA signatures in sporadic Creutzfeldt-Jakob disease are accompanied by alterations in miRNA silencing machinery and biogenesis

**DOI:** 10.1371/journal.ppat.1006802

**Published:** 2018-01-22

**Authors:** Franc Llorens, Katrin Thüne, Eulàlia Martí, Eirini Kanata, Dimitra Dafou, Daniela Díaz-Lucena, Ana Vivancos, Orr Shomroni, Saima Zafar, Matthias Schmitz, Uwe Michel, Natalia Fernández-Borges, Olivier Andréoletti, José Antonio del Río, Juana Díez, Andre Fischer, Stefan Bonn, Theodoros Sklaviadis, Juan Maria Torres, Isidre Ferrer, Inga Zerr

**Affiliations:** 1 Department of Neurology, University Medical School, Göttingen, Germany; 2 Center for Networked Biomedical Research on Neurodegenerative Diseases (CIBERNED), Barcelona, Spain; 3 German Center for Neurodegenerative Diseases (DZNE), Translational Studies and Biomarkers, Göttingen, Germany; 4 Centre for Genomic Regulation, Barcelona, Spain; 5 Prion Diseases Research Group, School of Health Sciences, Department Of Pharmacy, Aristotle University of Thessaloniki, Thessaloniki, Greece; 6 Department of Genetics, Development and Molecular Biology, School of Biology, Aristotle University of Thessaloniki, Thessaloniki, Greece; 7 Vall d'Hebron Institute of Oncology (VHIO), Barcelona, Spain; 8 German Center for Neurodegenerative Diseases (DZNE), Computational Systems Biology, Göttingen, Germany; 9 Centro de Investigación en Sanidad Animal (CISA-INIA), Madrid, Spain; 10 Institut National de la Recherche Agronomique/Ecole Nationale Vétérinaire, Toulouse, France; 11 Molecular and Cellular Neurobiotechnology, Catalonian Institute for Bioengineering (IBEC), Parc Científic de Barcelona, Barcelona, Spain; 12 Department of Cell Biology, University of Barcelona, Barcelona, Spain; 13 Molecular Virology group, Department of Experimental and Health Sciences, Universitat Pompeu Fabra, Barcelona, Spain; 14 German Center for Neurodegenerative Diseases (DZNE), Epigenetics and Systems Medicine in Neurodegenerative Diseases, Göttingen, Germany; 15 German Center for Neurodegenerative Diseases (DZNE), Tuebingen, Germany; 16 Center for Molecular Neurobiology University Medical Center Hamburg-Eppendorf, Hamburg, Germany; 17 Senior consultant, Bellvitge University Hospital-IDIBELL, Department of Pathology and Experimental Therapeutics, University of Barcelona, Hospitalet de Llobregat, Barcelona, Spain; Creighton University, UNITED STATES

## Abstract

Increasing evidence indicates that microRNAs (miRNAs) are contributing factors to neurodegeneration. Alterations in miRNA signatures have been reported in several neurodegenerative dementias, but data in prion diseases are restricted to ex vivo and animal models. The present study identified significant miRNA expression pattern alterations in the frontal cortex and cerebellum of sporadic Creutzfeldt-Jakob disease (sCJD) patients. These changes display a highly regional and disease subtype-dependent regulation that correlates with brain pathology. We demonstrate that selected miRNAs are enriched in sCJD isolated Argonaute(Ago)-binding complexes in disease, indicating their incorporation into RNA-induced silencing complexes, and further suggesting their contribution to disease-associated gene expression changes. Alterations in the miRNA-mRNA regulatory machinery and perturbed levels of miRNA biogenesis key components in sCJD brain samples reported here further implicate miRNAs in sCJD gene expression (de)regulation. We also show that a subset of sCJD-altered miRNAs are commonly changed in Alzheimer’s disease, dementia with Lewy bodies and fatal familial insomnia, suggesting potential common mechanisms underlying these neurodegenerative processes. Additionally, we report no correlation between brain and cerebrospinal fluid (CSF) miRNA-profiles in sCJD, indicating that CSF-miRNA profiles do not faithfully mirror miRNA alterations detected in brain tissue of human prion diseases. Finally, utilizing a sCJD MM1 mouse model, we analyzed the miRNA deregulation patterns observed in sCJD in a temporal manner. While fourteen sCJD-related miRNAs were validated at clinical stages, only two of those were changed at early symptomatic phase, suggesting that the miRNAs altered in sCJD may contribute to later pathogenic processes. Altogether, the present work identifies alterations in the miRNA network, biogenesis and miRNA-mRNA silencing machinery in sCJD, whereby contributions to disease mechanisms deserve further investigation.

## Author summary

miRNAs are small non-coding RNAs that regulate gene expression through complementary binding to their mRNA targets. Specific miRNA signatures have been proposed for several neurodegenerative diseases supporting the idea that miRNA deregulation is a common disease hallmark. Here we present the comprehensive miRNA signature in sporadic Creutzfeldt-Jakob disease (sCJD). Our study unravels the complex network of regional and disease-subtype miRNA alterations, and the presence of a disturbed miRNA biogenesis pathway and miRNA-mRNA silencing machinery. We also highlight the existence of time-dependent miRNA profiles and identify commonly regulated miRNAs between several dementias with cortical pathology sharing a partial clinical overlap and pathological involvement with sCJD. The present data shed light on the potential role of miRNAs as a contributing factor of pathogenic molecular traits associated with sCJD.

## Introduction

Creutzfeldt-Jakob disease (CJD) is a human transmissible spongiform encephalopathy characterized by behavior changes, progressive dementia, loss of coordination and myoclonus. At the molecular level, CJD is associated with the conversion of the normal, cellular prion protein (PrPC) to an abnormal conformation (PrPSc) and further accumulation of PrPSc in the brain in the form of protein aggregates [1]. Despite the established role of PrPC in several neuronal functions such as synaptic plasticity, neurotransmission and neuronal development, the molecular mechanisms triggering the PrPC to PrPSc conversion and the cellular pathways unchained by prion infection leading to neuronal damage and cell death remain elusive.

Sporadic CJD (sCJD) is the most common human prion disease, presenting a high degree of heterogeneity. sCJD is classified into six subtypes, based on variations at codon 129 of the prion protein gene (*PRNP* Met or Val) and on the size of protease resistant PrPSc (type 1 or 2). Among these subtypes MM1 and VV2 are the most prevalent [2,3] and give rise to unique clinical and neuropathological features, such as specific gliosis, neuroinflammation, spongiosis and synaptic loss signatures [2,4–7]. A widespread regional and subtype-specific mRNA and protein deregulation leading to the alteration of multiple biological functions and signaling pathways is also associated with sCJD [5,8–10].

Transcriptomic and proteomic patterns are regulated by several factors including miRNAs; these have been recognized as key regulators of gene expression. miRNAs are small (21–25 nucleotides long), non-coding RNAs, that regulate gene expression through partial complementary binding to their mRNA targets in the RNA-induced silencing complex (RISC). This miRNA-mRNA interaction usually leads to gene silencing through a variety of forms, including mRNA cleavage, translational repression and de-adenylation [11,12].

Several miRNAs are selectively expressed in the central nervous system (CNS) and have been reported to be involved in CNS development, function and pathogenesis [13,14]. In addition, specific miRNA signatures have been proposed for Alzheimer’s (AD), Parkinson’s (PD) and Huntington’s (HD) disease, as well as for Fronto-temporal dementia (FTD) [15–20], supporting the idea that miRNA deregulation is a common hallmark of neurodegenerative diseases. While the study of miRNAs in relation to prion pathogenesis has gained experimental momentum since several miRNAs were found to be altered in *in vivo* and *ex vivo* models of prion diseases [21–25], the miRNA signature in sCJD has not been reported so far.

A potential link between miRNAs and prion diseases has been suggested based on the co-localization of PrPC within RISC components in endosomes and multivesicular bodies. Binding of PrPC to the type III RNase Dicer (Dicer) and Argonaute (Ago) proteins, which represent essential components of the RISC loading complex, has been proposed as a requirement for effective repression of several miRNA targets [26]. Hence, miRNA deregulation could be triggered by many aspects of sCJD pathology, including replacement of the physiological PrP forms with pathological ones. Simultaneously, miRNA deregulation may have drastic consequences in sCJD gene expression patterns and may act as a contributing factor in the cascade of events leading to fast disease progression.

In order to increase our understanding of the miRNA contribution to sCJD pathogenesis, we performed small RNA-Sequencing (small RNA-Seq) in the two most affected brain regions in sCJD, frontal cortex (FC) and cerebellum (CB), in the two most prevalent sCJD subtypes (MM1 and VV2), which are linked to region specific clinical and pathological outcomes [2,27].

We demonstrate a strong regional and subtype-specific alteration of miRNA expression in sCJD and molecular alterations in miRNA biogenesis and silencing machinery; we further show that a subset of sCJD enriched miRNAs are actively incorporated in the RISC complex. Additionally, we detected the presence of commonly changed miRNAs in other neurodegenerative dementias such as AD, dementia with Lewy bodies (DLB) and fatal familial insomnia (FFI). Further, sCJD-related miRNA alterations were studied in a temporal manner, utilizing a sCJD mouse model; sCJD miRNA profiles were validated in the utilized animal model at clinical disease stages, whereas most of those miRNAs were not found to be regulated at earlier disease points suggesting diverse and dynamic miRNA expression programs during disease progression. Finally, we profiled selected miRNAs in the CSF of sCJD cases, which ruled out the presence of a major correlation between miRNA levels in CSF and brain tissue.

Altogether, our results show a significant deregulation of miRNA expression, activity and biogenesis in sCJD and they highlight the potential role of miRNAs in the pathology of prion diseases and alternative neurodegenerative conditions.

## Results

### Altered miRNA signatures in the FC and CB of sCJD MM1 and VV2 cases

**Fig 1:**
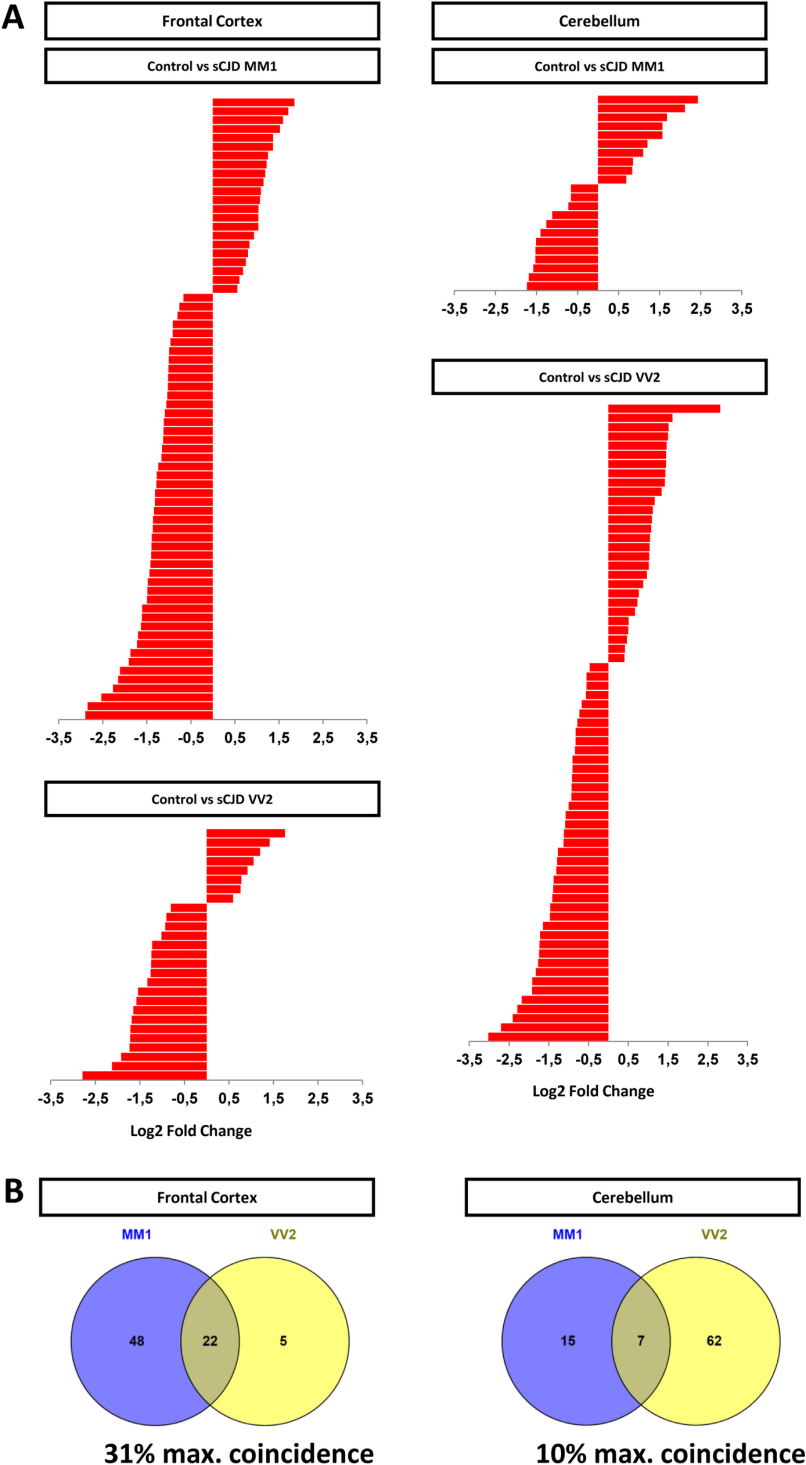
Small RNA-Seq analysis and miRNA expression levels in the FC and CB of sCJD. (A) Relative quantitation of regulated miRNAs in the FC and CB of sCJD MM1 and VV2 subtypes compared to age and gender matched controls by small RNA-Seq. For FC 13 controls, 11 sCJD MM1 and 13 sCJD VV2 were analyzed. For CB: 12 controls, 12 sCJD MM1 and 9 sCJD VV2 were analyzed. Differential expression in miRNA sequencing data was evaluated with the DESeq2 tool and significantly differently expressed miRNAs were detected according to an adjusted p value of <0.05. Data represent log2 fold change for each comparison. log2 fold change was >0.5 for upregulated miRNAs and <-0.5 for downregulated miRNAs. MA plots are also supplied for data visualization (S1 Fig). (B) Venn diagram of subtype-dependent altered miRNA in the FC and CB of sCJD by small RNA-Seq. Percentage of maximal coincidence (percentage of maximal number of miRNAs that can be coincident between groups) is indicated.

miRNA expression signatures were determined by small RNA sequencing in the frontal cortex (FC) and in the cerebellum (CB) of sCJD MM1 and VV2 and in age and gender matched controls. We selected these brain regions, because they are strongly affected in sCJD and display differential neuropathological patterns between MM1 and VV2 subtypes [2, 27]. Obtained sequences were annotated based on the overlap with publicly available genome annotations, including miRNAs, tRNAs, rRNAs, other small RNAs and genomic repeats. miRNAs represented an average of 27% of total counts (S1 Table). Total number of reads on the FC and CB mapping onto miRNAs with at least 2 counts in a given sample are shown (S2 Table). Two independent pipelines were used for the analysis of the differential miRNA expression, Seqbuster [28] and OASIS [29]. Both pipelines showed a high level of agreement in the detection of differentially expressed miRNAs (89%). Seqbuster analysis revealed the presence of 70 miRNAs with altered expression in the FC of sCJD MM1 and 27 in sCJD VV2 compared to controls (Fig 1A, S3 Table). In the CB, 22 miRNAs were changed in sCJD MM1 and 69 in sCJD VV2 compared to controls (Fig 1A, S3 Table). The majority of the differentially altered miRNAs were expressed in both tissues, suggesting that the changes on their levels are tissue specific (S2 and S3 Tables).

The miRNA signature in sCJD was highly dependent on the brain region and sCJD subtype (Fig 1B and S3 Table). Regarding sCJD subtype alterations, a high percentage of miRNAs were commonly regulated between both subtypes in the FC (31%) (Fig 1B). In the CB, the percentage of commonly altered miRNAs between subtypes was lower (10%) (Fig 1B).

### miRNA variability is ubiquitous in the brain of control and sCJD cases

Isoforms of a mature miRNA have been referred as isomiRs [30]. They are functionally active and highly abundant in brain tissue, both in control and in neurodegenerative diseases [15,31,32]. In the present study, 2883 and 4075 different isomiRs were found in the CB and FC, respectively (S4 Table). Furthermore, most of the sequences mapping onto miRNA database (reference miRNAs and IsomiRs) showed 3–50 counts. No major differences were detected between control and sCJD subtype cases regarding their isomiRs profiles (S2 Fig), suggesting that isomiR processing is not significantly altered in sCJD.

### sCJD-related miRNA validation and enrichment in RISC complexes

**Fig 2:**
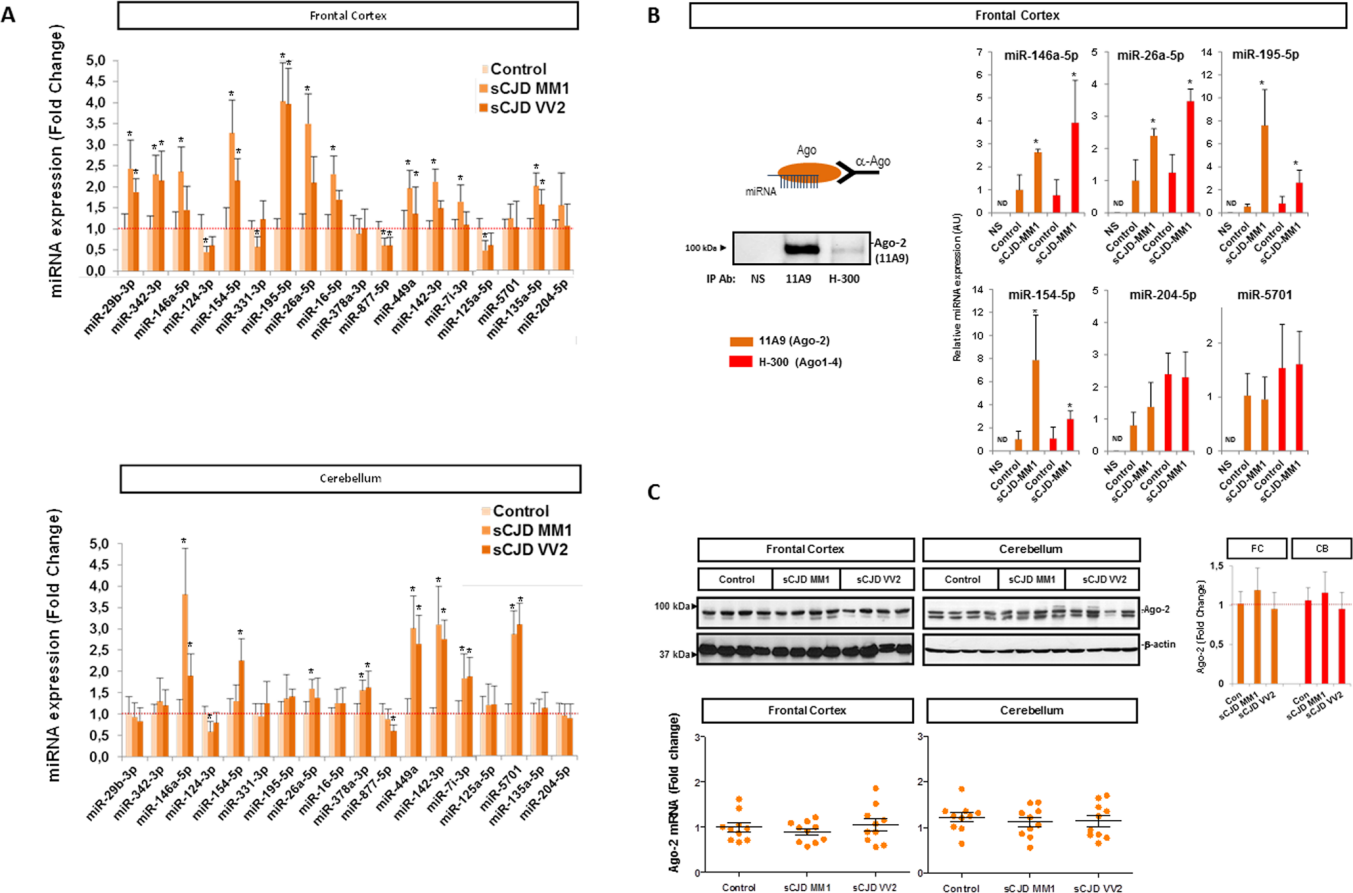
Regional and subtype-dependent miRNA expression confirmation and representative miRNA incorporation into functional complexes. (A) Validation of the small RNA-Seq signature in selected miRNAs by RT-qPCR analysis in the FC (upper panel) and CB (lower panel) of controls, sCJD MM1 and VV2. Results were normalized to the housekeeping genes U6 snRNA (figure) and RNU5 expression, which showed similar results in the expression analysis of deregulated miRNAs between control and sCJD cases. Housekeeping gene levels remained unaltered between groups. Normalization was performed relative to controls. Error bars indicate SD. (B) Detection of sCJD-related miRNAs by RT-qPCR in Ago immunoprecipitates from the FC of controls (n = 3) and sCJD MM1 brain homogenates (n = 3). The two Ago antibodies used (11A9 and H-300) were able to immunoprecipitate Ago-2 from brain tissue (left panel) and reported similar results in the specific enrichment of sCJD-related miRNAs (146a-5p, 26a-5p, 195-5p, 154-5p, 204-5p and 5701) in the FC of sCJD MM1 cases (right panels). miRNA-204-5p was selected as negative control, since no changes were detected between control and sCJD cases with small RNA-Seq and RT-qPCR analysis. Non-specific immunoglobulins (NS) were used as control antibody for the immunoprecipitation. Error bars indicate SD. (C) Upper panel: western blot analysis of Ago-2 (11A9 antibody) in the FC and CB control, sCJD MM1 and VV2 cases. Four representative cases per diagnostic group and brain region are shown in the western blot. Quantifications derived from densitometry analysis were performed in 15 cases per diagnostic group (n = 15/group). β-actin was used as loading control. Densitometries of the western blots are shown. Normalization was performed relative to controls. Error bars indicate SD. Bottom panel: Gene expression levels of Ago-2 in the FC and CB of controls, sCJD MM1 and VV2 cases by RT-qPCR. Results were normalized to housekeeping genes GAPDH (figure) and GUSB with similar results. Housekeeping levels remained unaltered between groups. 10 cases per diagnostic group (n = 10/group) were analyzed. In all cases, statistical significance (compared to controls) was set at *p<0.05.

A subset of miRNAs found to display altered expression in sCJD based on small RNA-Seq analysis was further validated by qPCR analysis. miRNAs were selected according to number of counts and fold change alterations in the RNA-seq analysis and/or their previous association with prion disease pathogenesis and/or other neurodegenerative diseases [21,23,33,34]. A total of 18 miRNAs were analyzed in both regions. The alterations in the levels of 15 and 10 miRNAs were validated in a regional specific manner in the FC and CB, respectively (Fig 2A). In sCJD FC, miRNAs 29b-3p, 342-3p, 146a-5p, 154-5p, 195-5p, 26a-5p, 16-5p, 449a, 142-3p, let7i-5p and 135a-5p were increased, while miRNAs 124-3p, 331-3p, 877-5p and 125a-5p were decreased compared to controls, in agreement with RNA-seq data. miRNAs 378a-3p and 5701, which expression was only altered in CB did not present changes in the FC. In CB, miRNAs 146a-5p, 154-5p, 26a-5p, 378a-3p, 449a, 142-3p, let7i-3p and 5701 were increased, and miRNAs 124-3p and 877-5p were decreased in sCJD, in agreement with RNA-seq data. The rest of miRNAs, which expression was only altered in FC did not present changes in the CB. Finally, miRNA-204-5p, a miRNA presenting no alterations in the FC and CB of sCJD by RNA-seq analysis, was used as negative control showing no changes among groups at qPCR level. Therefore, while most of the qPCR validated miRNAs were altered in both disease subtypes, a subset of them presented subtype-specific changes, which were in agreement with small RNA-Seq data (S3 Table).

To confirm that upregulated miRNAs in sCJD brain tissue were functionally active, we performed RISC immunoprecipitations in the FC of control and sCJD MM1 cases using two different Argonaute (Ago) antibodies detecting Ago-2 (11A9) and Ago1-4 family members (H-300). These antibodies were able to immunoprecipitate Ago-containing miRNA complexes from brain tissue (Fig 2B). RNA extraction from immunoprecipitates and further qPCR analysis allowed us to detect miRNA enrichment for a subset of miRNAs with increased expression in sCJD MM1 brain tissue according to qPCR analysis (miRNA-146a-5p, miRNA-26a-5p, miRNA-195-5p and miRNA-154-5p). As negative controls, miRNA-204-5p and miRNA-5701 were tested. According to RNA-seq and qPCR data, miRNA-204-5p was regulated neither in the FC nor in the CB of sCJD, while miRNA-5701 was upregulated only in the CB of sCJD cases. In agreement with this, miRNA-204-5p and miRNA-5701 levels were unchanged in RISC immunoprecipitates between control and sCJD cases (Fig 2B). To rule out the possibility that the differences in RISC-miRNA enrichment were due to alterations in Ago-2 expression between controls and sCJD, Ago-2 levels were analyzed by qPCR and western blot. No alterations were found in Ago-2 protein and mRNA levels between controls and sCJD cases, either in the FC or in the CB (Fig 2C). Similarly, no changes on the expression levels of GW182, an Ago binding protein essential for miRNA-mediated gene silencing [35] were detected between control and sCJD cases (S3A and S3B Fig).

### Alterations in miRNA-mRNA silencing complexes in sCJD

**Fig 3:**
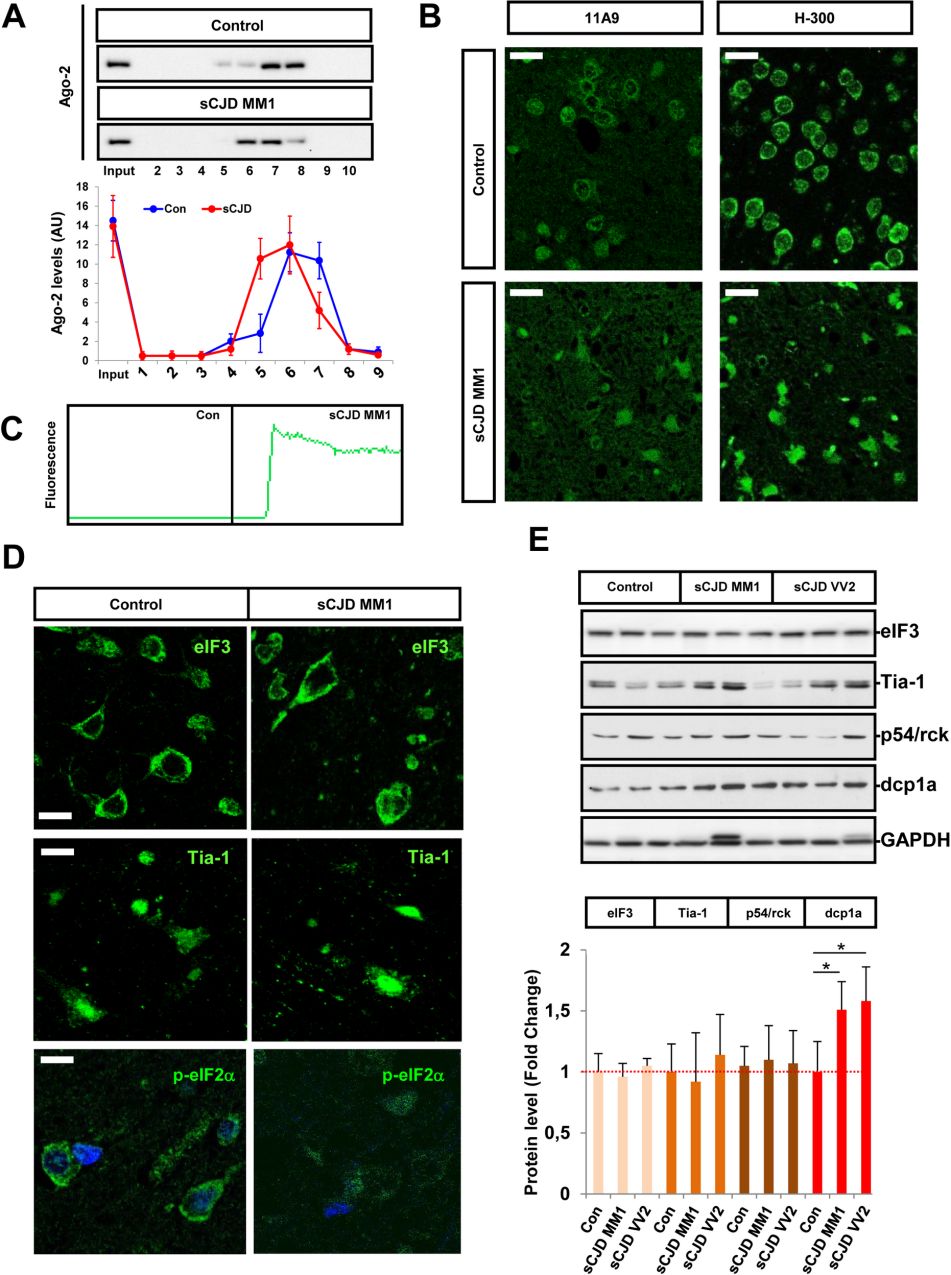
Alterations in the levels and distribution of the miRNA silencing machinery in sCJD. (A) Western blot analysis for Ago-2 immunodetection (upper panel) and densitometry (lower panel) of the chromatography gel filtration fractions from the FC of control (n = 3) and sCJD MM1 (n = 3) cases. Western blot analysis shows a representative image of a control and a sCJD case, while densitometry shows mean values and SD of all cases studied. (B) Representative fluorescence photomicrographs of Ago immunoreactivity in the FC of control and sCJD MM1 cases. The two antibodies used (11A9 and H-300) showed similar Ago distribution between control and sCJD MM1 cases. Scale bar = 25μm (C) RT-QuIC analysis of Ago-2 immunoprecipitates (11A9) obtained from the FC of brain homogenates of control (n = 3) and sCJD MM1 (n = 3) cases. RT-QuIC assays were run in triplicate for each sample. A representative curve for each condition is shown. (D) Representative fluorescence photomicrographs of eIF3, Tia-1 and p-eIF2α(Ser51) immunoreactivity in the FC of control and sCJD MM1 cases. Scale bar for eIF3 = 10 μm and Tia-1 = 20μm, for p-eIF2α(Ser51) = 10μm (E) Western blot analysis of eIF3, Tia-1, p54/rck and dcp1a in the FC of control, sCJD MM1 and VV2 cases. Three representative cases per diagnostic group and brain region are shown in the western blot. Quantifications derived from densitometry analysis were performed in 15 cases per diagnostic group (n = 15/group). GAPDH was used as a loading control. Densitometries of the western blots are shown. Normalization was performed relative to controls. Error bars indicate SD. In all cases, statistical significance (compared to controls) was set at *p<0.05.

Next, we investigated potential alterations in the miRNA-mRNA silencing complexes that could explain the alterations in sCJD miRNA signatures previously detected. As a first step, we performed gel filtration chromatographic assays of control and sCJD brain homogenates. Ten fractions containing different proteomic patterns according to their molecular weight were obtained. PrP levels were homogeneously distributed along the chromatographic fractions as described before [36,37] (S4A and S4B Fig). Western blot analysis revealed the presence of Ago-2 in higher molecular weight fractions in sCJD compared to control samples, suggesting that Ago-2 in sCJD is interacting with a different subset of partners (Fig 3A). This is in agreement with a different subcellular localization of Ago in sCJD brain tissue as revealed by immunohistochemistry analysis. Indeed, using two different antibodies, we detected an increased nuclear localization of Ago in the FC of sCJD MM1 and VV2 cases, in contrast to controls, where staining was mainly detected in the cytoplasmic compartment (Fig 3B and S5A and S5B Fig). While Ago-2 expression was mainly detected in neurons, both in controls and sCJD cases, double immunofluorescence analysis revealed the presence of Ago-2 positive microglial cells (S5 Fig). In contrast, no differences on subcellular localization were detected for GW182 between control and sCJD cases (S3 Fig).

PrP and Ago-2 are interacting proteins in physiological conditions [26], but nothing is known about the potential role of PrPSc in the RISC complex. Since the endosomal compartment, in which RISC assembly and turnover occurs, has been proposed as a site of prion conversion [38] we aimed to investigate the presence of PrPSc in Ago-2 complexes, which could be one of the contributors to their altered chromatographic Ago-2 patterns in sCJD. To this end, Ago-2 immunoprecipitates from the FC of controls and sCJD MM1 were subjected to RT-QuIC analysis. Positive signal was detected in immunoprecipitates from sCJD samples, but not from controls (Fig 3C), indicating the presence of pathogenic PrP in Ago-2 complexes, in agreement with the presence of RT-QuIC signal and protein oligomers in Ago-2 containing chromatographic fractions (S4C and S4D Fig).

The alteration of RISC components in sCJD and the well-known presence of reticulum stress in models of prion disease [39–41] raised the possibility that stress granules (SG), which are normally transient structures, form stable complexes in sCJD. Immunohistochemical and immunoblot analysis of the SG markers eukaryotic initiation factor 3 (eIF3) and T-cell-restricted intracellular antigen-1 (Tia-1) revealed that, in sCJD, neither their subcellular localization nor their expression levels were altered (Fig 3D and 3E). In agreement with this, we did not detect hyper-phosphorylation of the SG activator eIF2α in sCJD (Fig 3D). Similarly, levels of the SG and p-bodies marker DEAD-Box Helicase 6 (p54/rck) showed no alterations between controls and sCJD. However, increased expression levels of the specific p-bodies marker decapping mRNA 1a (dcp1a) was detected in sCJD MM1 and VV2 (Fig 3E). Altogether, our findings suggest the presence of alterations in the p-bodies-dependent mRNA decay mechanisms without the activation of stress granule responses.

### Altered miRNA biogenesis machinery in sCJD

**Fig 4:**
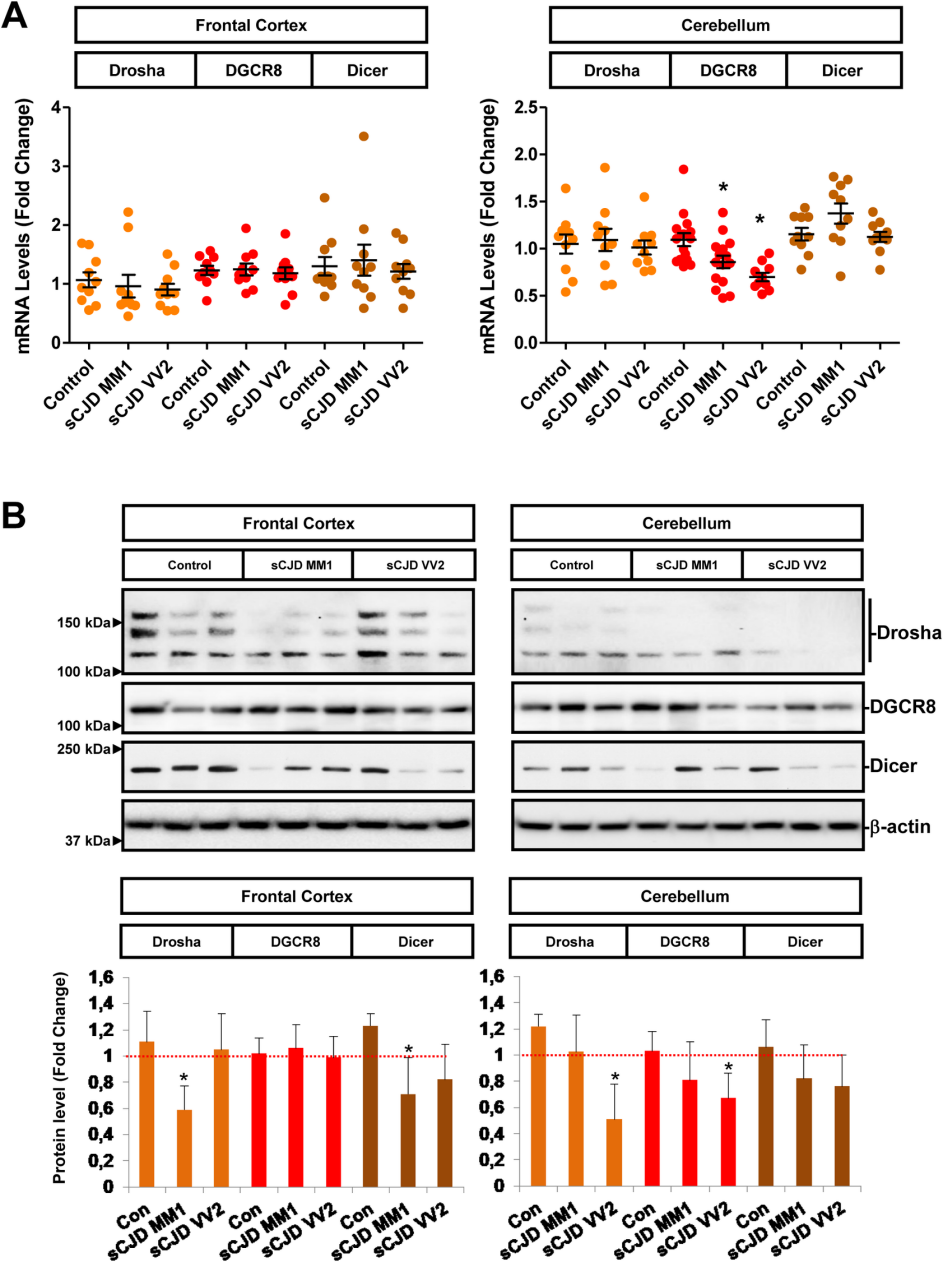
Altered expression levels of miRNA biogenesis components in sCJD. (A) Gene expression levels of Drosha (n = 10), DGCR8 (n = 10) and Dicer (n = 10) in the FC and CB of controls, sCJD MM1 and VV2 cases by RT-qPCR. Results were normalized to housekeeping genes GAPDH (figure) and GUSB with similar results. Housekeeping levels remained unaltered between groups. (B) Protein levels of Drosha, DGCR8, Dicer in the FC and CB of controls, sCJD MM1 and VV2 cases, by western blot analysis. Three representative cases per diagnostic group and brain region are shown in the western blot. Quantifications derived from densitometry analysis were performed in 15 cases per diagnostic group (n = 15/group). β-actin was used as a loading control. Densitometries of the western blots are shown. Normalization was performed relative to controls. Error bars indicate SD. In all cases, statistical significance (compared to controls) was set at *p<0.05.

Disruption of the miRNA biogenesis pathway components might cause alteration of miRNA homeostasis and neurodegeneration [42,43]. miRNA alterations in sCJD prompted us to consider possible alterations in the miRNA biogenesis pathway. The expression levels of three key components of miRNA biogenesis, the ribonucleases Dicer and Drosha and the microprocessor complex DGCR8, a cofactor of Drosha, were studied in sCJD brain samples. mRNA expression analysis revealed decreased DGCR8 levels in the CB of sCJD cases (Fig 4A). At the protein level, Drosha levels were significantly lower in the FC of sCJD MM1 and in the CB of sCJD VV2, resembling the regional and subtype pathological involvement of the disease. Decreased Dicer levels were detected in the FC of sCJD MM1, while reduced levels of DGCR8 were found in the CB of sCJD VV2 (Fig 4B). The absence of direct regional and/or subtype-specific alterations among the main components of the miRNA biogenesis pathway suggests the presence of a complex impairment of the miRNA biogenesis machinery in sCJD. Additionally, we investigated the expression levels of Exportin 5, a RanGTP-dependent dsRNA-binding protein mediating pre-miRNAs nuclear export [44,45], which expression is deregulated in AD, but not in PD or Down’s syndrome dementia [46]. Exportin 5 levels in sCJD were altered neither at mRNA nor at protein levels compared to controls (S6 Fig).

### Neural-type miRNA expression profiling in sCJD

**Fig 5:**
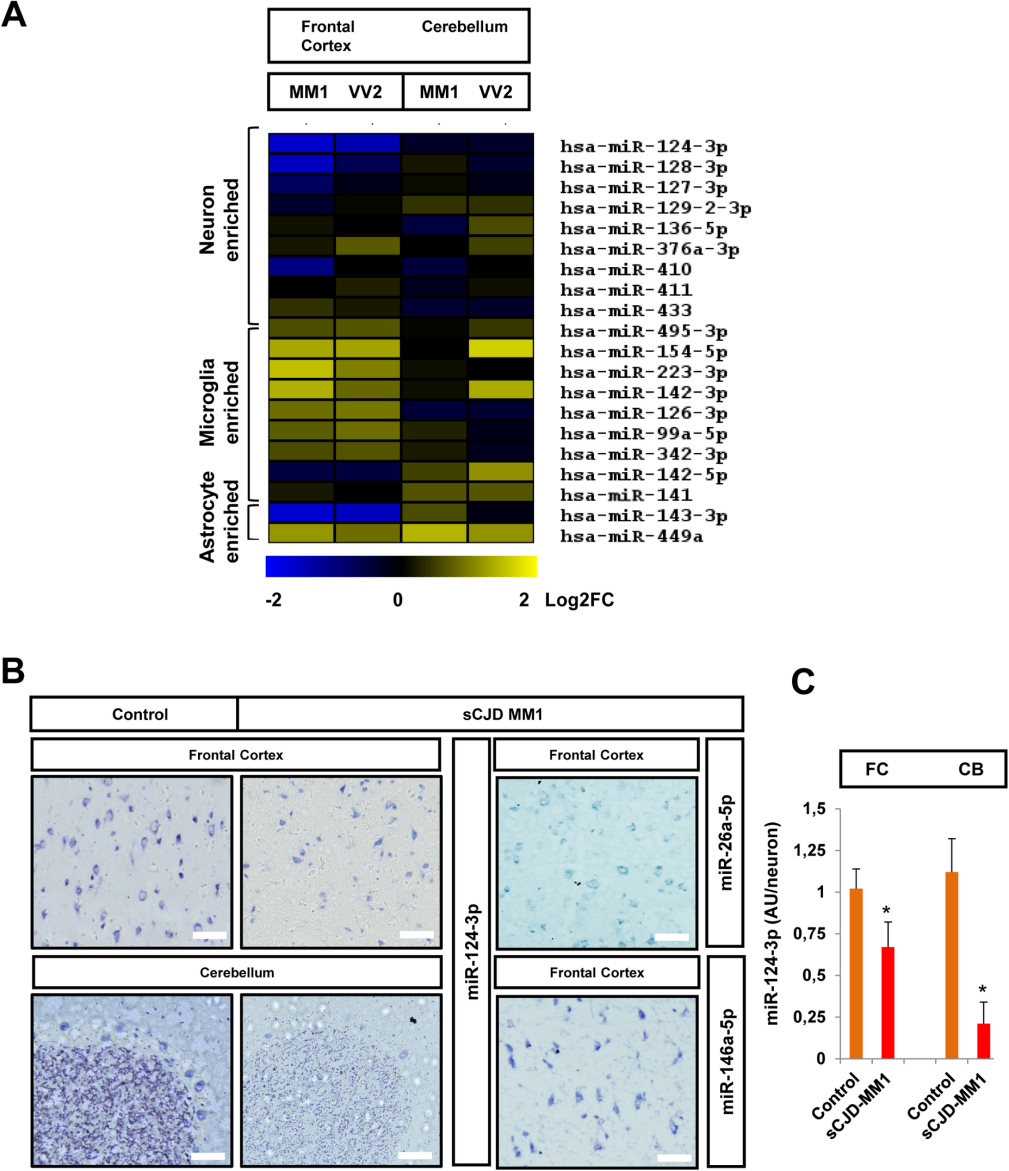
Neural-type miRNA profiling in sCJD. (A) Heat map analysis of the neuron, microglia and astrocyte enriched miRNAs, whose levels were changed in the FC and CB of sCJD MM1 and VV2 cases by RNA-seq analysis. Neural-type enriched miRNAs were reported in the bibliography based on neural-type enrichment analysis (49). (B) In situ hybridization of miRNA-124-3p in the FC and CB of control and sCJD MM1 brain tissue, and of miRNAs 26a-5p and 146a-5p in the FC of sCJD MM1 brain tissue. (C) Quantification of miRNA-124-3p intensity in the FC and CB of control and sCJD MM1 neurons. >100 neurons in total were quantified for each group. Normalization was performed relative to controls. AU/neuron indicates arbitrary units quantified in the densitometry analysis for each neuron. Error bars indicate SD. Scale bar in FC = 30μm and in CB = 50μm. In all cases, statistical significance (compared to controls) was set at *p<0.05.

A prominent hallmark in sCJD pathogenesis is the concomitant increase of neuronal loss and gliosis [2,47]. Since cell-type specific miRNA signatures have been described in neural populations [34,48,49], we aimed to investigate the neural-type miRNA expression profiling in sCJD. First, sCJD-regulated miRNAs were compared to those reported to be enriched in neurons, microglia and astrocytes [34]. The expression of neuron-enriched miRNAs was not significantly altered in sCJD (Fig 5A), indicating that the sCJD-related miRNA signature is not a mere consequence of neuronal death. On the other hand, most microglia and astrocyte-enriched miRNAs presented deregulated levels in sCJD, most likely as a result of glial proliferation and activation. To gain insight into subcellular and neural-type localization of sCJD-associated miRNAs in human brain tissue, *in situ* hybridizations were performed for the following miRNAs: miRNA-124-3p, miRNA-26-5p and miRNA-146a-5p (Fig 5B and 5C). The three miRNAs were localized in the cytoplasm of neurons. Additionally, miRNA-146a-5p labeling was also detected in capillary walls and in some small cells compatible with glial morphology. Decreased miRNA-124-3p staining detectable in sCJD was associated with a reduced number of neurons. However, we also detected less signal intensity in surviving sCJD neurons, both in the FC and in the CB (Purkinje and granular cells) regions (Fig 5B and 5C), in agreement with the idea that lower expression of neuronal-related miRNAs in sCJD is not exclusively associated with neuronal loss.

### Cross-validation of sCJD-miRNA signature in alternative neurodegenerative diseases

**Fig 6:**
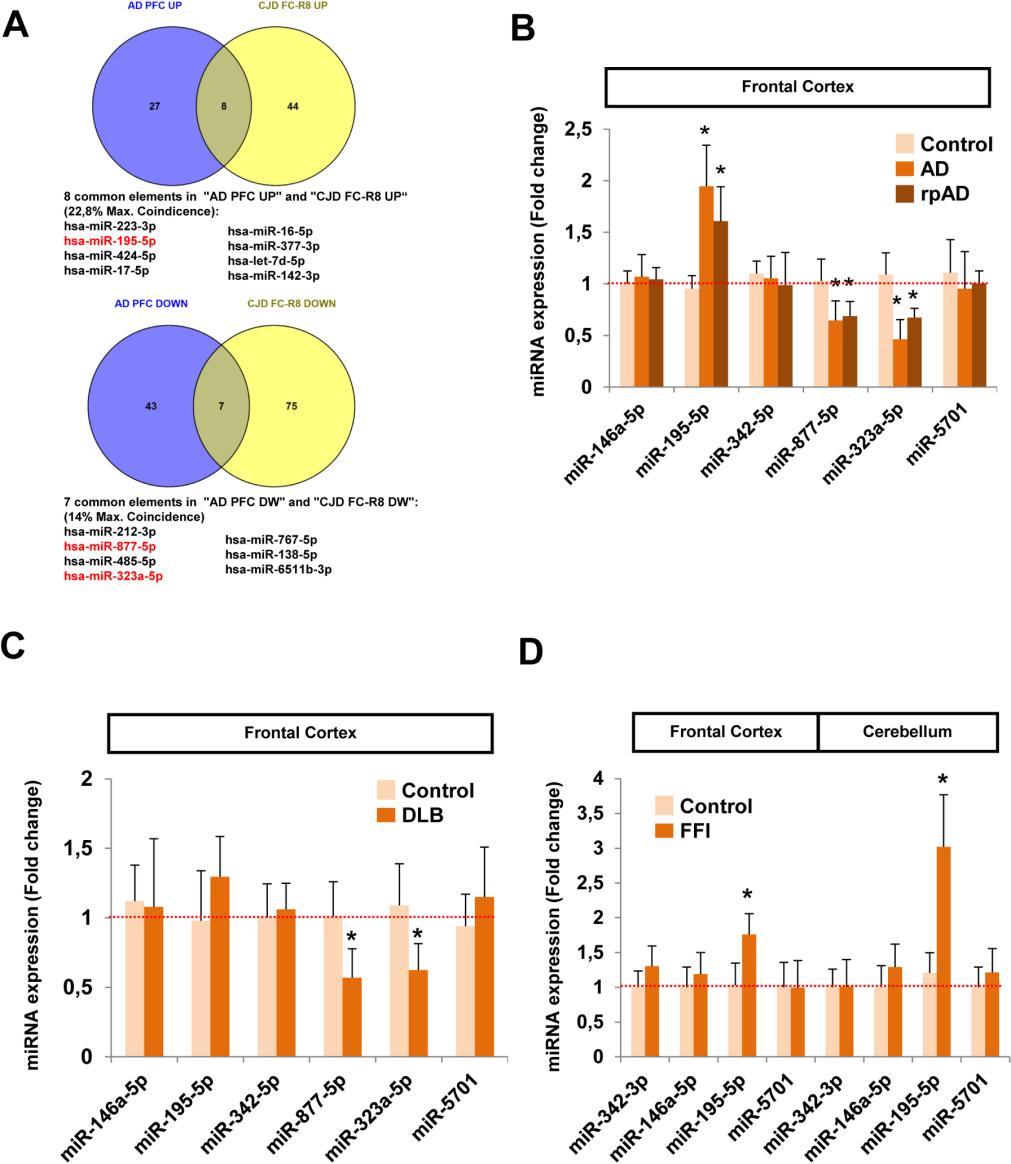
Analysis of commonly altered miRNAs in sCJD, AD, DLB and FFI. (A) Venn diagrams of the comparison between altered miRNAs in AD and sCJD. Commonly deregulated miRNAs in the PFC of AD cases obtained from small RNA-seq analysis (20) (blue circles) and in the FC of sCJD cases obtained from small RNA-seq analysis in the present work (yellow circles). Common elements and percentage of maximal coincidence between groups are shown. Among these, the deregulated expression levels of miRNA-195-5p, miRNA-877-5p and miRNA-323a-5p (marked in red) were previously validated by qPCR in the FC of sCJD cases (Fig 2). (B) RT-qPCR analysis for miRNA-146a-5p, miRNA-195-5p, miRNA-342-5p, miRNA-877-5p, miRNA-323a-5p and miRNA-5701 in the FC of controls (n = 5), AD (n = 8) and rpAD (n = 6) cases. (C) RT-qPCR analysis for miRNA-146a-5p, miRNA-195-5p, miRNA-342-5p, miRNA-877-5p, miRNA-323a-5p and miRNA-5701 in the FC of controls (n = 5) and DLB (n = 5) cases. (D) RT-qPCR analysis for miRNA-342-5p, miRNA-146a-5p, miRNA-195-5p and miRNA-5701 in the FC and CB of controls (n = 3) and FFI cases (n = 3). Results were normalized to the housekeeping U6 snRNA expression, which remained unaltered between groups. Normalization was performed relative to controls. Error bars indicate SD. In all cases, statistical significance (compared to controls) was set at *p<0.05.

Several neurodegenerative disorders share pathological hallmarks such as accumulation of protein aggregates and self-propagation, and common pathways seem to contribute to the neurodegenerative mechanisms in different diseases [50–52]. Thus, we speculated that an overlap between miRNA sCJD profiling and other dementia-related conditions could exist. In order to select the most appropriate miRNAs we compared the miRNA signatures in the FC of sCJD obtained from the small RNA-seq analysis from this study with the one reported in the pre-frontal cortex (PFC) of AD cases by Lau et al. [20]. Eight (22.8% max. coincidence) and seven miRNAs (14% max. coincidence) were commonly increased and decreased respectively in both datasets (Fig 6A). Among these, miRNA-195-5p, 877-5p and 323a-5p were previously validated in sCJD from our small RNA-Seq dataset and miRNA-195-5p and 877-5p were validated by qPCR (Fig 2A).

To perform a cross-validation study with the corresponding sCJD brain regions and methodologies we extracted RNA from the FC of AD and DLB cases and age-matched controls. Six miRNAs were selected: i) miRNA-195-5p, 877-5p and 323a-5p, commonly regulated in the FC of sCJD and in the PFC of AD, ii) miRNA-146a-5p and miRNA-342-3p, reported to be altered in AD and prion disease models [22,23,53,54] and iii) miRNA-5701. The latter was used as a negative control due to its exclusive enrichment in the CB of sCJD. In AD samples, we detected coincident gene expression regulations with sCJD for miRNA-195-5p (increased) and for miRNA-877-5p and 323a-5p (decreased) (Fig 6B). Rapid progressive forms of AD (rpAD) mimicking the disease progression and cognitive decline of sCJD [55,56] were included in our study. No significant differences in the expression levels of the six analyzed miRNAs were detected between AD and rpAD (Fig 6B). In DLB, we detected coincident gene expression regulations with sCJD for miRNA-877-5p and miRNA-323a-5p (both with decreased expression levels) (Fig 6C).

Finally, we extended our analysis to cases of fatal familial insomnia (FFI), a genetic prion disease presenting mild cortical and cerebellar affection [57,58]. A subset of four sCJD-regulated miRNAs in the FC and CB were tested. Only miRNA-195-5p showed common expression profiles in sCJD and FFI, with increased expression in both brain regions compared to age-matched control (Fig 6D) indicating a lack of complete specificity of miRNA patterns between neurodegenerative diseases from same etiology.

### Temporal dependent sCJD miRNA profiling

**Fig 7:**
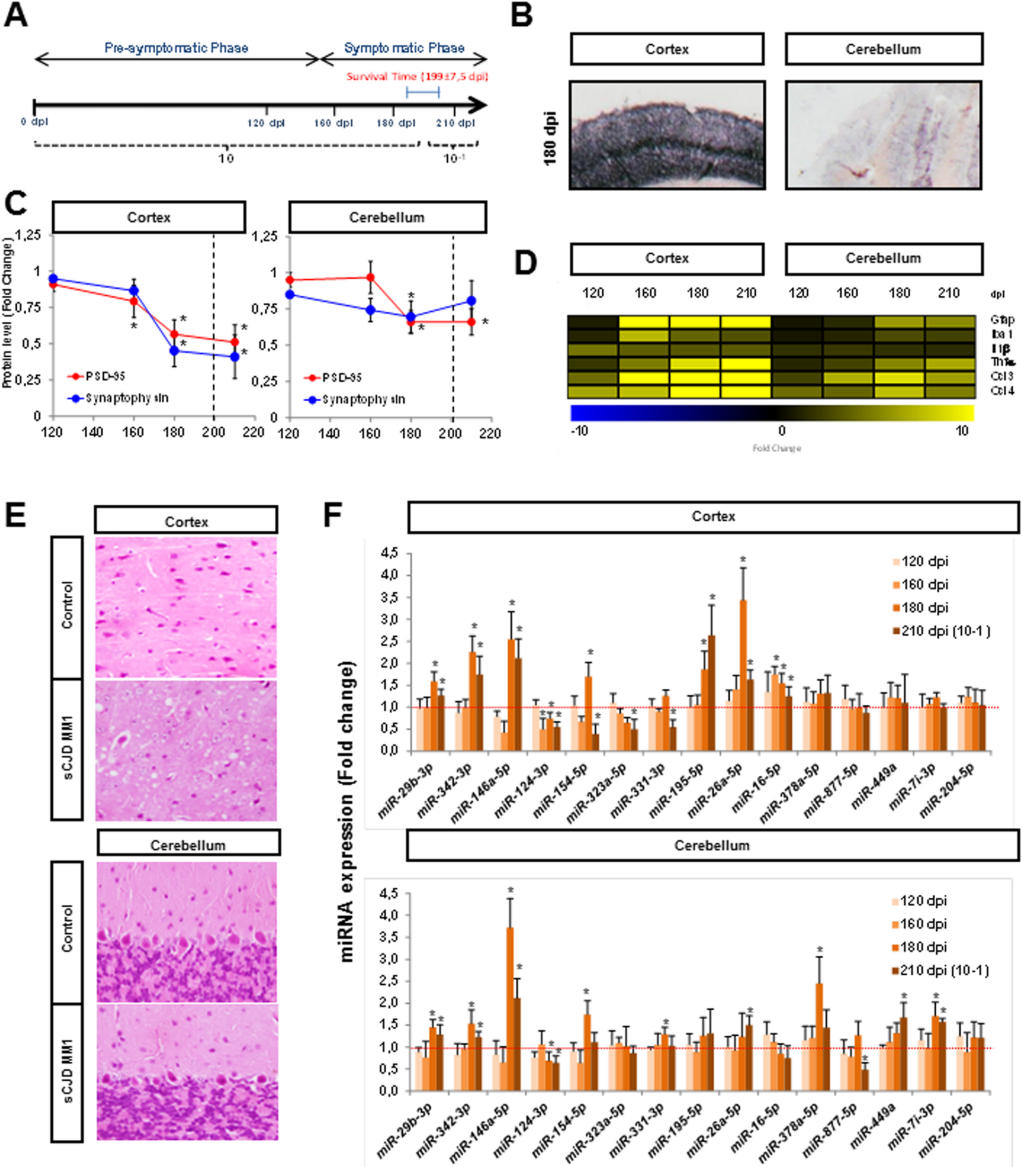
Regional and temporal-dependent neuropathological characteristics and miRNA signatures in the sCJD MM1 mouse model tg340-PRNP129MM. (A) tg340 mice were inoculated with control or sCJD MM1 homogenates; cortex and CB samples were collected at different time points: 120 dpi for pre-symptomatic phase and 160 dpi, 180 dpi and 210 dpi for symptomatic phase (n = 4–5 per group). Animals sacrificed at 210 dpi were inoculated with a 10–1 inoculum dilution. (B) PET-blot analysis for the detection of PrPSc in the cortex and CB of sCJD MM1 inoculated tg340 mice at clinical disease stage. (C) Densitometric analysis of western blots developed for PSD-95 and synaptophysin in the cortex and CB of tg340 samples at different disease stages. Significant alterations on PSD-95 and synaptophysin levels between control and sCJD inoculated animals is indicated. Statistical significance was set at *p<0.05, (n = 4–5 per group). (D) Heat map analysis of key inflammatory mediators and cytokines measured with RT-qPCR analysis in the cortex and CB of control and sCJD MM1 inoculated tg340 mice at different stages of the disease. Fold change between sCJD MM1 infected and control animals is shown. (E) Hematoxylin-eosin staining in the cortex and CB of control and sCJD MM1 infected tg340 animals. (F) RT-qPCR analysis of the miRNAs validated in human sCJD tissue in the cortex and CB of tg340 mice. Samples from different time points of disease progression were analyzed. Fold change between sCJD MM1 infected and control animals is shown. Results were normalized to the housekeeping gene U6 snRNA expression. U6 levels remained unaltered between groups. Normalization was performed relative to controls. Error bars indicate SD. In all cases, statistical significance (compared to controls) was set at *p<0.05.

To gain insights into the temporal-dependent sCJD miRNA profiles we took advantage of the sCJD MM1 mouse model tg340-PRNP129MM (tg340) inoculated with sCJD MM1 brain homogenate. Mice were sacrificed at pre-clinical (120 dpi), early clinical (160 dpi) and clinical (180 dpi and 210 dpi) disease stages. Survival time was 199 ± 7.5 days (Fig 7A). To confirm the disease-specific regional and subtype neuropathological and biochemical alterations in the tg340 mice, several prion hallmarks were assessed. Increased PrPSc deposition (Fig 7B), synaptic damage (Fig 7C and S7 Fig), neuroinflammation (Fig 7D) and spongiform degeneration (Fig 7E) were detected in the cortex compared to the CB of the tg340 infected mice. These data confirmed the region specific alterations of sCJD MM1 subtype in the tg340, resembling the most prominent cortical pathology in human sCJD MM1 [5,59]. Next, the expression levels of the qPCR-validated miRNAs in sCJD were analyzed in a temporal manner (Fig 7F). Ten and eleven miRNAs were validated in the cortex and in the CB at one of the clinical stages (180 and/or 210 dpi), respectively. Among these, seven were commonly changed in both regions, in agreement with data from sCJD MM1. An interesting observation from our qPCR panel was the temporal specific alterations of the sCJD-related miRNAs, since only miRNA-16a-5p (increased) and miRNA-124-3p (decreased) showed altered levels at early clinical stages of the disease in the cortex (Fig 7F and S8 Fig). This indicates that qPCR validated miRNAs are reflecting late pathogenic alterations, while miRNA-16a-5p and miRNA-124-3p may also participate in early pathogenic mechanisms. In agreement with this, functional enrichment analysis from small RNA-Seq indicates that the main common functions related to the sCJD-regulated miRNAs are cell death and survival (S5 Table).

These results suggested that diverse deregulated miRNA, rather than a specific miRNA deregulation, could contribute to the pathological mechanisms in sCJD. Therefore, we highly purified miRNAs from the FC of control, sCJD MM1 and VV2 brains and transfected them into neuroglioma (H4) and differentiated neuroblastoma (SH-SY5Y) cells. Five miRNAs were analyzed with qPCR in both cell lines, resembling the disease subtype profiling in human sCJD brain (S9A Fig). Overexpression of sCJD-MM1 purified miRNAs lead to an increased cell death in neuroblastoma, but not in neuroglia cells (S9B Fig), indicating that the overexpression of the sCJD regulated miRNAs is able to induce subtype specific cell death in neuron-like cells. Finally, to gain insight into the potential upstream regulators of differential miRNA expression in sCJD a motif enrichment analysis was performed for data generated from FC of sCJD MM1 sequencing. Among the known sCJD related pathways, our analysis revealed a significant enrichment of a STAT3-binding motif for miRNAs with increased expression in sCJD (S6 Table). This suggests that the expression of sCJD-specific miRNAs is under STAT3 regulation, which has been described as activated not only in experimental models of prion diseases [60,61], but also in sCJD post-mortem tissue [5].

### Differential miRNA expression levels of abundant CSF miRNAs in sCJD

**Fig 8:**
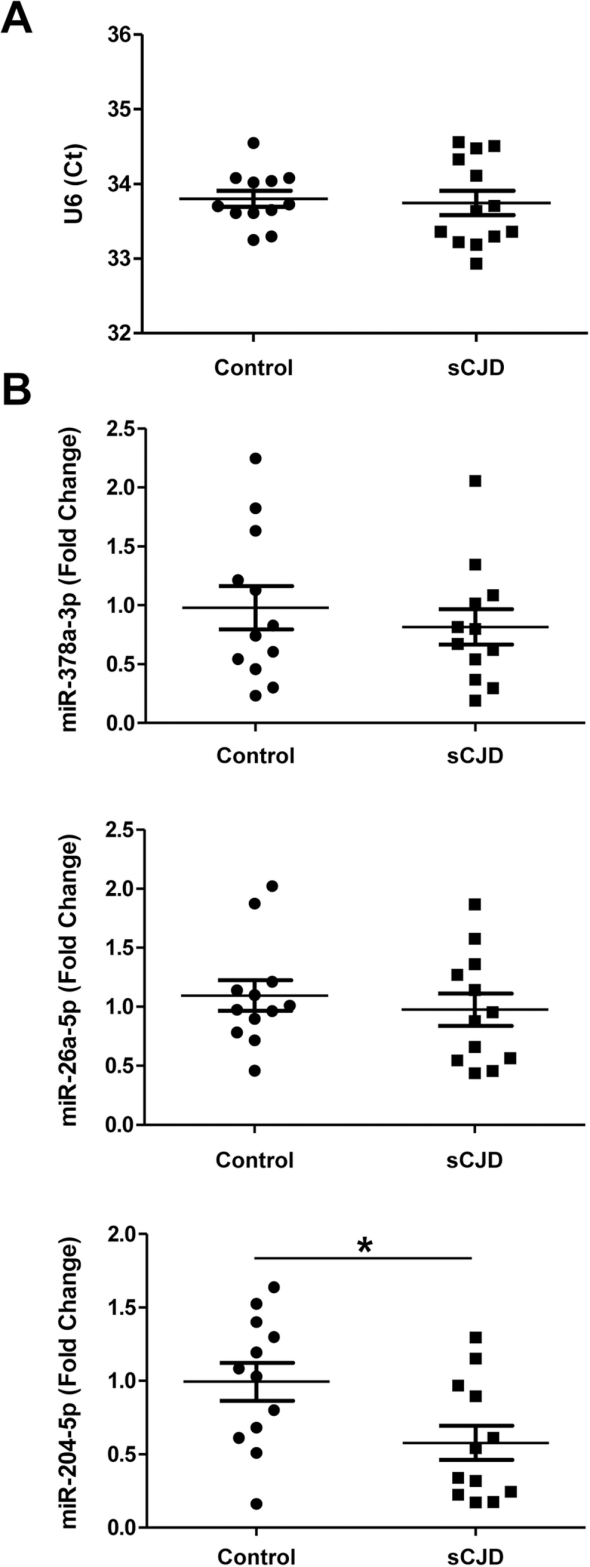
miRNA profiling in the CSF of control and sCJD cases. RT-qPCR analysis of the housekeeping U6 snRNA (A) and miRNA-378a-3p, miRNA-26a-5p and miRNA-204-5p (B) in the CSF of control (n = 12) and sCJD cases (n = 12). Samples were normalized by the relative expression of the housekeeping small nuclear RNA U6, which showed stable Ct values between the studied groups. Statistical significance was set at *p<0.05.

CSF miRNAs have been suggested as a source of biomarkers in neurodegenerative disease mirroring alterations in the brain tissue [62,63]. Thus, we aimed to investigate whether the detected miRNA alterations in the brain tissue of sCJD patients could be reflected in the CSF. CSF RNA was extracted from twelve control and twelve sCJD cases and was subjected to qPCR analysis for the following miRNAs: 154-5p, 204-5p, 378a-3p, 331-3p, 26a-5p, 195-5p, 124-3p, 7i-3p, 143-3p, 449a and 5701. For normalization we used the non-coding small nuclear RNA U6, which showed stable levels between control and sCJD cases (Fig 8A). Detectable signal (35<Cts) was obtained for miRNAs 378a-5p, 26a-5p and 204-5p, with miRNA-204-5p showing significantly decreased levels in sCJD compared to controls (Fig 8B).

## Discussion

In the present study, we report the first systematic analysis of miRNA populations in two brain areas and two disease subtypes of sCJD cases, utilizing small RNA-seq analysis. We detected marked alterations in miRNA patterns with the presence of regional and sCJD-subtype specific signatures, with highest deregulations in the FC of MM1 and in the CB of VV2 cases, two brain regions and subtypes showing high pathological affection in sCJD [1]. In this regard, the low overlap between sCJD subtypes altered miRNAs in CB and FC could be explained by the singular pathology of sCJD VV2 in CB, where characteristic PrPSc aggregates (synaptic and plaque-like) and degree of spongiform degeneration, neuronal loss and neuroinflammation profiling are detected [5–7], following classical well-known sCJD regional and subtype-dependent molecular neuropathology [2,47,64].

Small RNA-seq provides a blind and unbiased approach to the study of the small RNA transcriptome. However, a limitation of this technique is the presence of potentially biased fold changes when number of counts is low. Therefore, confirmatory analysis by qPCR is indispensable to consistently validate the regulation of selected targets. The enrichment of regulated miRNAs in Ago-containing complexes, as well as a severe, global reduction of miRNA expression levels in sCJD compared to controls described herein, supports the idea that alterations in expression levels are translated into the functional miRNA silencing machinery. Small RNA-seq also revealed length and sequence heterogeneity for the vast majority of miRNAs. However, the fact that the proportion of different miRNA variants detected by isomiR profiling was similar in all cases indicates that the molecular mechanisms involved in isomiR generation are not altered in sCJD, similarly to the situation previously reported for HD [15].

Data on miRNA alterations in sCJD are limited to two targeted studies, including very small cohorts of cases. Upregulation of miRNA-146a-5p in the neocortex of sCJD cases (n = 3 sCJD, n = 3 controls) [65] and upregulation of miRNA-342-3p in sCJD brain tissue (n = 2 sCJD, n = 1 control) [23] are in agreement with our observations. In contrast, several studies have been devoted to analysing the miRNAome in prion animal models. A lack of major correlation between regulated miRNAs in sCJD and scrapie-infected mice was detected in high-throughput studies. However, miRNAs: 146a-5p, 342-3p, 142-3p, 26a-5p, 124a-3p (RNA-seq altered and qPCR validated in sCJD) and 338-5p, 18a-3p, 455-5p, 182-5p (RNA-seq altered in sCJD) are commonly altered in at least one of the studies where scrapie miRNA profiles have been investigated [21–23,25,33,66]. Additionally, two miRNAs upregulated in the *basis pontis* of bovine spongiform encephalopathy-infected macaques, miRNA-342-3p and miRNA-494-3p [23], were also enriched in sCJD, but exclusively in the FC region. Although low co-occurrence on miRNA profiles may be due to different methodologies, the absence of detailed regional studies in mouse models and the specific prion-related pathology in humans may explain this divergence. Altogether, these data highlight the importance of detailed regional and disease-subtype studies in prion diseases. Yet, intra-species comparisons are now achievable through the study of humanized *PRNP* mouse models, which not only fully recapitulate pathological hallmarks of human disease [5,59] but also, as reported in the present study, resemble human miRNA profiling. In this regard, alterations in miRNA signatures in tg340 at clinical stages are not detectable at pre-clinical stages. This finding, along with the presence of enriched miRNA-target genes related to cell death and survival, indicates that sCJD-regulated miRNAs may play a role in the molecular mechanisms related to the neurodegenerative process and that a different population of miRNAs, would be responsible for the primary causative events of the disease.

Relative decreased mature miRNA levels in sCJD are consistent with decreased expression levels of Dicer, Drosha and DGCR8. The miRNA biogenesis pathway is highly conserved and its disruption is a well-reported cause of neurodegeneration. Loss of Dicer levels provokes neuronal dysfunction in Purkinje cells [67], dopaminergic neurons [68] and motor neurons [69], and increased excitability of CA1 pyramidal neurons [70] while compromising axonal integrity in Schwan cells [71]. In addition, Dicer protein levels have been found to be decreased in temporal lobe epilepsy patients with hippocampal cell loss, with about half of the miRNAs in the tissue displaying reduced levels [72]. Finally, similar to our observations, impaired miRNA biogenesis at Dicer level associated with downregulation of miRNA levels and reorganization of Dicer and Ago-2 complexes has recently been described in amyotrophic lateral sclerosis [73] suggesting that miRNA malfunction could be a contributor to pathogenesis associated with protein-misfolding associated diseases. Interestingly, lack of expression changes in Exportin 5 suggests that changes detected in the miRNA expression profiles in sCJD are most likely not due to alterations in the nuclear export of pre-miRNA.

Whether alterations of miRNA biogenesis and homeostasis in sCJD are primary factors in the neurodegenerative phenotype of the disease due to dysfunctional miRNA maturation, or are a consequence of the pathology, deserves further studies.

Besides highlighting alterations in miRNA biogenesis and network, our results also reveal a remodelling of the miRNA-mRNA silencing complexes in sCJD. This is sustained by partial re-distribution of Ago-2 to higher molecular weight chromatographic fractions, increased Ago nuclear reactivity, presence of Ago-2 positive microglial cells and increased expression of p-body marker dcp1 in sCJD brain tissue. Ago-2 and RISC components have recently been found in the nucleus of humans and Drosophila and associated in multi-protein complexes with functional silencing activity over nuclear targets [74,75], as well as with additional functions in pre-mRNA splicing and transcriptional repression [75]. The role of Ago proteins, especially Ago-2, in prion pathology deserves attention not only due to its differential localization in sCJD, potentially altering its physiological functions, but also because PrPC has been described as an Ago-2 interacting partner, promoting the accumulation of miRNA-RISC effector complexes [26]. PrPC is internalised into the endocytic recycling pathway and most of the molecules are recycled intact to the cell surface [76]. Since late endosomes and/or multivesicular bodies are the main site for intracellular conversion of PrPC to PrPSc, and RISC formation and/or turnover depends on the endosomal pathway [77,78]; the detection of PrPSc in Ago-2 complexes provides a link between RNA silencing and membrane trafficking in sCJD pathogenesis. Additionally, a translocation of Ago-2 from cytoplasm to nuclear fractions in sCJD would alter, and potentially impair, the silencing of cytoplasmic mRNA targets by the RISC complex.

As additional modifiers of the miRNA-mRNA silencing complexes in sCJD we investigated the presence of SG, which appear in the cell under stress conditions such as oxidative and endoplasmic reticulum stress [79,80], two well-known sCJD hallmarks [41,81]. Although both p-bodies and SG may support overlapping cellular functions and share components, they are not equivalent and they are spatially distinct. SG are thought to be responsible for mRNA storage as these sites lack the decapping enzyme [82], and their formation is mediated through phosphorylation of eIF2α and aggregation of Tia-1 [83,84]. Neither increased eIF2α phosphorylation nor altered levels of SG markers were detected, suggesting that SG are not specific structures in sCJD.

A surprising finding of our study was the absence of massive reduction of neuronal-enriched miRNAs levels, indicating high miRNA stability in brain after neuronal death. An exception to this was the decreased levels of miRNA-124-3p, also decreased in experimental models of prion diseases [21,22,66]. *In situ* hybridization supports the idea that miRNA-124-3p is not merely decreased as a consequence of neuronal death, since surviving neurons express less miRNA-124-3p compared to those from controls, pointing towards a specific role for this miRNA in sCJD pathology. miRNA-124-3p is the most abundant miRNA in the brain and promotes neuronal differentiation and maintenance of neuronal identity [85]. In AD, its expression is decreased in the anterior temporal cortex [86], decreased in the dentate gyrus, and upregulated in the locus coeruleus [87]. In an *ex vivo* model of PD, miRNA-124-3p is decreased regulating apoptosis and impaired autophagy [88]. This plethora of evidence denotes a functional role of miRNA-124-3p in neurodegeneration.

In fact, our study details the existence of common miRNA traits in the cortical region of AD, DLB and sCJD. There is virtually no information on the functions of the two commonly regulated miRNAs in the brain of the three dementias (miRNA-877-5p and miRNA-323a-5p). On the contrary, miRNA-195-5p, elevated in AD and sCJD, downregulates Aβ production by targeting APP and BACE1, and protects against chronic brain hypoperfusion-mediated dementia [89]. Its overexpression also led to reduced BACE1 and decreased Aβ levels in an independent study [90]. Although the precise role of miRNA-195-5p in prion diseases is unknown, we also detected increased levels in the FC of fatal familial insomnia (FFI), a genetic prion disease with moderate cortical involvement [91].

Based on the conception that one miRNA can target multiple mRNAs and one mRNA can be targeted by multiple miRNAs, our results support multiple lines of evidence indicating that the result of the intricate alterations in miRNA networks and clusters, rather than representing a change in the expression of a single miRNA, are responsible for pathological phenotypes [92–94]. Thus, the precise miRNA homeostasis in sCJD brain would underlie the spectrum of molecular and phenotypic cues, in agreement with the acquisition of a disease-related phenotype by a neuronal-like cell line upon transfection with the sCJD-associated miRNA transcriptome.

CSF miRNAs may reflect alterations in brain pathology of neurodegenerative diseases. Indeed, miRNA profiling in AD and PD correlates with disease status and pathological features [63,95] but less is known about the levels of brain-regulated miRNAs in the CSF. Our targeted study revealed that only miRNAs with reported high CSF expression levels were detectable [96]. Of these, only miRNA-204-5p displayed decreased levels in sCJD cases. Interestingly, this miRNA was not statistically regulated in sCJD brain. Our findings are in line with those reported in AD, where no obvious relationship between altered miRNAs in CSF and pathologically affected brain regions was found [17,95]. As the reason for this lack of correlation is unknown, it is tempting to speculate that different disease stages between CSF and brain sample collection (time of diagnosis for CSF versus post-mortem for brain tissue) may contribute to these differences. Additionally, as the origin of CSF miRNAs is not well understood, CSF miRNAs may originate not only from brain, but also from extracraneal tissues.

In summary, our study presents, for the first time, comprehensive miRNA signatures in human prion diseases and unravels the complex network of regional and disease-subtype miRNA alterations in sCJD, as well as revealing the presence of a disturbed miRNA biogenesis pathway and miRNA-mRNA silencing machinery. It also highlights the existence of time-dependent miRNA profiles along disease duration and identifies commonly altered miRNAs between several dementias sharing a partial clinical overlap. Taken together, the present data shed light on the potential role of miRNAs as a contributing factor and/or transmitters of pathogenic molecular traits in sCJD.

## Materials and methods

### Reagents

List of Taqman probes assays, Exiqon primer sets and antibodies is given in S7 Table. Lipofectamine 2000 was from Thermo Fisher Scientific. Thioflavin and Propidium Iodide were from Sigma. WST-1 was from Roche. TruSeq Small RNA Sample Preparation Kit was from Illumina. CHROMA SPIN-200 spin columns were from Clontech and Protein G magnetic beads were from Invitrogen.

### Cases and general processing

Brain tissue processing has been described before [5–7,97]. Mean ages and gender for studied control and sCJD cases, for RNA-seq, qPCR and western blot analysis are detailed in S8 Table. Information on the mean ages and gender for the AD, DLB and FFI samples that were analysed with RT-qPCR in the present study are as below: For AD analysis: Control = 75 (3M/2F), AD = 76 (4M/4F) and rapid progression AD (rpAD) = 77 (3M/3F). rpAD cases were AD cases with disease duration shorter than 2 years. For DLB analysis: Control = 71 (3M/2F), DLB = 75 (3M/2F). For FFI analysis: Control = 58 (2M/1F), FFI = 52 (2M/1F). Biochemical studies including sCJD and FFI cases were performed in biosafety rooms (S3 level). mRNA and miRNA levels were associated neither to RNA integrity number (RIN) values nor to post-mortem time. Protein levels were not associated to post-mortem time.

CSF samples were obtained from an unrelated series of patients with sCJD and from controls. sCJD patients diagnosed with probable or definite sCJD according to established criteria were considered for the sCJD group [98]. The control group was composed of patients suffering from neurological conditions (S9 Table). The presence of neurodegenerative diseases in the control cohort was excluded in the follow-up clinical diagnostic, and CSF neurodegenerative biomarkers (14-3-3, tau, p-tau and Aβ42) were negative at the time of diagnosis.

### CJD subtype characterization

*PRNP* codon 129 genotyping (Met or Val) was performed after genomic DNA isolation from blood samples according to standard methods [99]. Western blot PrPSc profile was classified as type 1 (un-glycosylated PrPSc at 20 kDa) or type 2 (un-glycosylated PrPSc at 19 kDa), based on electrophoretic mobility after proteinase K (PK) digestion [4,64].

### RNA extraction and sequencing

The purification of RNA from FC and CB of CJD and age-matched controls was performed using the mirVana isolation kit (Ambion, US) according to the manufacturer’s instructions. After purification, samples were treated with the RNase-free DNase set (Ambion, US) for 30 min to avoid carry over and subsequent amplification of genomic DNA. The concentration of each sample was determined using the NanoDrop 2000 spectrophotometer (Thermo Scientific, US). RNA integrity number (RIN) was verified with the Agilent 2100 Bioanalyzer (Agilent, US). The threshold for further sample selection was set to RIN value equal to or greater than 5.5. Starting from 1 μg of total RNA, libraries were prepared following the TruSeq Small RNASample Preparation Guide protocol from Illumina (Part # 15004197 Rev. E). Library quality was assessed on the Agilent Technologies 2100 Bioanalyzer. DNA was loaded into a lane of a single-read flow cell at a concentration of 3–3.5 pM for cluster generation using a single-read cluster generation kit (Illumina). From 13 to 15 barcoded samples were sequenced per lane. The sequencing primer (5′-CGACAGGTTCAGAGTTCTACAGTCC GACGATC-3′) was annealed to the clusters and the flow cell was then mounted on a Hiseq 2000 instrument (Illumina) for sequencing, and 36–41 sequencing cycles were performed. A PhiX control lane loaded at a concentration of 2 pM was used to monitor run quality. Image processing and base calling was performed using Illumina sequencing analysis pipelines v0.3.0 or v1.3.2. A total of 72 samples were analyzed by small RNA-seq: For FC 13 controls, 13 sCJD MM1 and 13 sCJD VV2 were analyzed for CB: 12 controls, 12 sCJD MM1 and 9 sCJD VV2.

### Small RNA data processing and mapping

Reads were trimmed to 36 nt and ligation adapters were removed using the adrec.jar program from the seqBuster suite (http://github.com/lpantano/seqbuster) [28] with the following options: java -jar adrec.jar 1 8 0.3. Sequences were mapped to the hg19 genome with the command line: bowtie -f -v 1 –a–best–strata. For summing up miRNA read counts we mapped the reads against miRBase version 21 hairpins with the miraligner.jar tool with these options: java–jar miraligner.jar 1 3 3.

Out of a total of several million reads, we discarded any reads without a minimum of 10-nt linker subsequence directly adjoining the insert, showing two or less mismatches. Then sequences were mapped to human pre-miRNA and mature miRNA databases provided in the miRBase (http://miRNA.sanger.ac.uk/sequences/, Release 14), as well as mRNA, ncRNA, repeats and genome databases available at (http://hgdownload.cse.ucsc.edu/goldenPath/hg18/bogZips/), using Mega BLAST.

For motif discovery, deregulated miRNAs were searched for the genomic coordinates of their primary miRNAs. Upstream regions of 1kb size from each miRNA were extracted and exported to BED files and the script findMotifsGenome.pl in HOMER suite was used to find transcription factor binding motifs within promoter regions using genome assembly hg38 [100].

For differential expression analysis we used DESeq2 analysis and log2 transformation of the count data. Padj value was <0.15 and nominal p values were in all cases <0.05. We used the count matrix generated by Seqbuster. Only miRNAs consistently expressed (counts > 10) in at least 10 samples out of the 21–26 were included in each analysis (controls versus MM1 or VV2 cases).

### Analysis of miRNA variability (IsomiRs)

IsomiRs were annotated and analyzed using the SeqBuster tool [28]. For miRNA annotation the following parameters were configured: one mismatch, 3 nts in the 3’ or 5’-trimming variants, 3 nts in the 3’-addition variants. These options permitted annotations of the following types of alignment: (i) perfect match, where the sequence is completely identical to the reference sequence; (ii) trimming at the 3’-end of the reference miRNA sequence, which is an miRNA variant several nucleotides shorter or longer that matches to the mature or precursor reference sequence, respectively; (iii) trimming at the 5’-end of the sequence, an analogous case as to (ii) but focused on the 5’-end of the miRNA; (iv) nucleotide additions at the 3’-end of the sequence and (v) nucleotide substitutions, showing nucleotide changes with respect to the reference sequence. The parameters for the alignment to the mRNA and genome databases allowed up to one mismatch and up to three nucleotide additions in the 3’-terminus.

For deep characterization of IsomiRs we applied several filters. First, the sequences considered in the analysis presented a frequency above 3. Second, 10 was chosen as the ‘Contribution Cut-Off’ parameter, meaning that every isomiR considered in the analysis contributes by more than 10% to the total number of variants annotated in the same miRNA locus. Third, we applied the Z-score option to exclude sequencing errors as the possible cause of the nucleotide changes observed in some variants.

### miRNA purification for transfection experiments

RNA samples extracted with the mirVANA isolation kit (Ambion) using the specific protocol for small RNA isolation were run on a 6% Urea–PAGE. The bands containing miRNAs (15–30 nt) were excised from the gel and incubated for 1 hour with 250 mM NaCl and 1 mM EDTA. miRNAs were then precipitated with 2.5 volumes of 100% ethanol (v/v) over night at −80°C, washed twice with 70% ethanol and re-suspended in nuclease-free water.

### Cell cultures and transfection

H4 and SH-SY5Y cells (American Type Culture Collection) were cultured at 37°C in a 95%/5% Air/CO2 water-saturated atmosphere in Dulbecco’s modified Eagle’s medium (DMEM, Thermo Fisher Scientific) containing 10% heat inactivated fetal bovine serum (FBS, Thermo Fisher Scientific), 2 mM L-glutamine and 100U/ml Penicillin/streptomycin (Gibco). SH-SY5Y cells were differentiated with DMEM containing 3% FBS and 10 μM all-trans retinoic acid (RA, Sigma) for 72 hours. Differentiation medium was replenished after 48 hours. Cells were transfected with 250 ng of highly purified miRNAs with Lipofectamine 2000 (Invitrogen) following the manufacturer’s instructions.

### Gel filtration

Analysis of protein fractions according to their molecular weight was performed as described before [36] using CHROMA SPIN-200 (Clontech, USA) spin columns. Columns were pre-spun at 200xg for 3 min to remove storage buffer. Buffer exchange was made by the addition of 500 μl lysis buffer followed by centrifugation at 200 xg for 3 min. This step was then repeated. 75 μl of 1% brain homogenates were applied to the gel bed. After spinning at 120 xg for 2 min elution fractions were collected, and 40 μl of extraction buffer was added after each centrifugation step.

### sCJD MM1 mice–tg340 PRNP129MM

The tg340 mouse line expressing about 4-fold level of the human PrP M129 on a mouse PrP null background was generated as described elsewhere [59]. Control and sCJD MM1 brain tissues (10% (w/v) homogenates) were used as inocula. Individually identified 6–10 week-old mice were anesthetized and inoculated in the right parietal lobe using a 25-gauge disposable hypodermic needle. Additionally, MM1 inoculum dilutions were performed to study prolonged disease times; animals were sacrificed at 210 dpi (10–1 dilution). Mice were observed daily and their neurological status was assessed weekly. The animals were euthanized at pre-symptomatic (pre-clinical: 120 dpi) and symptomatic (early clinical: 160 dpi and clinical: 180 dpi) stages and the brain was removed. A part of the brain was fixed by immersion in 10% buffered formalin, to quantify spongiform degeneration and perform immunohistological analysis. The other part was frozen at −80°C, for extraction of protein and RNA. Paraffin-embedded tissue blots from tg340 mice samples was carried out as described previously [5]. For each tissue sample, serial sections, 4 mm thick for PET blot, were collected on membranes. Serial sections were stained with hematoxylin and eosin. SHa31 antibody was used for PrP immunodetection.

### RT-qPCR

In order to confirm the direction of the miRNA alterations detected by RNA-seq in sCJD cases (increased or decreased levels compared to controls) by an independent methodology qPCR analysis of selected miRNAs was performed. Quantitative real time PCR for miRNAs was performed using the miRCURY LNAmiRNA PCR System (Exiqon) following Minimum Information for Publication of Quantitative Real-Time PCR Experiments guidelines. RNA was extracted with the mirVana isolation kit (Ambion) following the manufacturer’s instructions. PCR amplification and detection were performed with the Roche LightCycler 480 detector, using 2x SYBR GREEN Master Mix. The reaction profile was: Polymerase Activation/Denaturation (95°C for 10 min) followed by 40 amplification cycles (95°C-10 sec, 60°C-20 sec). miRNA levels were calculated using the LightCycler 480 software. Samples were normalized by the relative expression of the housekeeping small nuclear RNAs U6 and U5. Housekeeping genes showed no variability between analyzed groups. CT values obtained from the miRNA qPCR analysis ranged from 18 to 31.

Quantitative real time PCR for mRNAs was performed using Taqman probes (Life Technologies) on total RNA extracted with mirVana’s isolation kit (Ambion) following the manufacturer’s instructions. PCR assays were conducted in duplicate using cDNA samples obtained from the retrotranscription reaction and diluted 1:15 in 384-well optical plates. PCR amplification and detection were performed with the Roche LightCycler 480 detector, using Taqman Universal PCR Master Mix, following the manufacturer’s instructions. The reaction profile was as follows: denaturation–activation cycle (95°C for 10 min) followed by 40 cycles of denaturation–annealing–extension (95°C, 10 min; 72°C, 1 min; 98°C, continuous). mRNA levels were calculated using the LightCycler 480 software. Samples were normalized based on the relative expression of a housekeeping gene (glyceraldehyde-3-phosphate dehydrogenase [GAPDH]). The housekeeping gene showed no variability between analyzed groups.

### Western blot

Human tissues were lysed in lysis buffer: 100 mM Tris pH 7, 100 mM NaCl, 10 mM EDTA, 0.5% NP-40 and 0.5% sodium deoxycolate plus protease and phosphatase inhibitors. After centrifugation at 14,000g for 20 min at 4°C, supernatants were quantified for protein concentration (BCA, Pierce), mixed with SDS-PAGE sample buffer, boiled, and subjected to 8–15% SDS-PAGE. Gels were transferred onto nitrocellulose membranes and processed for specific immunodetection by chemiluminescence (ECL Amersham, US) using the indicated antibodies. Densitometries were carried out with the ImageJ software and values were normalized using β-actin levels.

### RISC immunoprecipitation

Protein G magnetic beads were pre-equilibrated in lysis buffer (150 mM NaCl, 50 mM Tris, 0.5% NP40, protease and phosphatase inhibitors) and mixed with 1 mg of human FC brain homogenate from either control or sCJD MM1 cases and with 4 μg of Ago antibodies 11A9 or H-300. As a control, 4 μg of an unspecific IgG antibody was used. Complexes were incubated overnight at 4°C with gentle end-to-end shaking. To extract the immunoprecipitated RNA, beads were washed three times in lysis buffer and resuspended in phenol-chloroform. The RNA in the aqueous phase was precipitated for 1 h at -80°C after addition of 2.5-fold volume ethanol and 0.1-fold volume NaAc (3mol/l); precipitated RNA was pelleted by centrifugation for 25 min at 4°C at 20,000xg. After washing in cold 70% ethanol, centrifugation and air drying, RNA was re-suspended in 10 μl of RNase-free water. The miRCURY LNA Universal RT miRNA PCR kit (Exiqon) was used for miRNA reverse transcription. For this, 6.5 μl of re-suspended RNA was applied in a total RT reaction volume of 10 μl (2μl 5x reaction buffer, 1μl enzyme mix, 0.5μl nuclease free water). cDNA was synthesized as described before for miRNA RT. A 1:80 cDNA dilution was used for miRNA quantification via real-time PCR amplification and miRNA LNA primer sets.

### miRNA in situ hybridization

For miRNA recognition locked nucleic acid (LNA) modified probes combined with signal amplification technology using enzyme-labeled immunoassay were obtained from Exiqon (Vedbaek, Denmark). The following double digoxigenin (DIG)-labelled sequences of the LNA probes were used: hsa-miRNA-124: 5’-DIG/ggcattcaccgcgtgcctta/DIG-3’, hsa-miRNA-146a 5’-DIG/aacccatggaattcagttctca/DIG-3’, has-miRNA-26a 5’-DIG/agcctatcctggattacttgaa/DIG-3’. The sequence of the U6 snRNA positive control probe was: 5’-DIG/cacgaatttgcgtgtcatcctt/-3’. 6 μM-thick brain tissue sections were deparaffinised, deproteinized with Proteinase K (15μg/ml) at 37°C for 10 min, washed in PBS and dehydrated in increasing concentrations of ethanol. Probe hybridization was performed over night at 55°C with 100 nM (hsa-miRNA-146a, hsa-miRNA-26a), 40 nM (miRNA-124), or 1 nM (U6 snRNA) of LNA probe diluted in hybridization mix. After stringent washing in salt sodium citrate (SSC) buffer and blocking with 2% sheep serum/1% bovine serum albumin, probe-target complex was visualized immunologically with anti-DIG antibody (Roche, 1:800) conjugated to alkaline phosphatase acting on the chromogen NBT/BCIP. In some cases, slides were counterstained with nuclear fast red (Vector laboraties). For quantification of miRNA-124-3p, 3 controls and 2 sCJD MM1 cases were used.

### Immunofluorescence

For immunofluorescence analysis in brain tissues, de-waxed sections, 4 microns thick, were stained with a saturated solution of Sudan black B (Merck, DE) for 15 min, to block the auto-fluorescence of lipofuscin granules present in cell bodies, and then rinsed in 70% ethanol and washed in distilled water. The sections were boiled in citrate buffer to enhance antigenicity and blocked for 30 min at room temperature with 10% fetal bovine serum diluted in PBS. Then, the sections were incubated at 4°C overnight with primary antibodies. After washing, the sections were incubated with Alexa488 or Alexa546 (1:400, Molecular Probes, US) fluorescence secondary antibodies against the corresponding host species. The sections were mounted in Immuno-Fluore mounting medium (ICN Biomedicals, US), sealed, and dried overnight. Sections were examined with a Leica TCS-SL confocal microscope.

### Real time quaking induced conversion (RT-QuIC)

RT-QuIC was performed as previously described [101] with minor modifications. Briefly, recombinant PrP (10 μg) was seeded with 15 μl of Ago-2-Immunoprecipitates in 85 μl of reaction buffer. Reaction was set in a final volume of 100 μl and placed in a 96-well black optical bottom plate (Fisher Scientific). Each sample was run in duplicate. Prepared plates were sealed and incubated in a FLUO Star OPTIMA plate reader (BMG Labtech Ortenberg, GE) at 42°C for 80 h, with intermittent shaking cycles consisting of 1 min double orbital shaking at the highest speed (600 rpm) followed by a 1 min break.

### CSF analysis

Lumbar punctures were performed for diagnostic purposes at the time point of the first diagnostic work-up and samples were stored at −80°C until analysis. 14-3-3 protein was analyzed as described previously [102] and total tau was quantitatively measured using the enzyme-linked immunosorbent assay kits INNOTEST-hTAU-Ag from Fujirebio according to the manufacturer’s instructions. RT-QuIC analysis was performed as described before [101]. RNA purifications from CSF were performed using miRCURY RNA Isolation Kit–Biofluids (Exiqon) following manufacturer-provided protocol with minor modifications. 200 μl CSF input volume was used and treated with 2 μg/μl Proteinase K in order to optimize the RNA yield. As an inert RNA carrier 2 μg Glycogen per CSF sample was added. The miRCURY LNA Universal RT miRNA PCR kit (Exiqon) was used for miRNA reverse transcription. For this, 4 μl of re-suspended RNA was applied as input in a total RT reaction volume of 10 μl (2 μl 5x reaction buffer, 1 μl enzyme mix, 3 μl nuclease free water) and cDNA was synthesized as described before for miRNA RT. A 1:80 cDNA dilution was used for miRNA quantification via real-time PCR amplification and miRNA LNA primer sets. The small RNA U6 revealed to be stable in CSF samples from sCJD and control samples and was used as reference gene for miRNA quantification.

### Statistical analysis

For comparisons of the two groups, the Mann-Whitney test was used. In multiple comparisons, the Kruskal-Wallis test was used. Dunn's multiple comparison test was used for *post hoc* analysis. Statistical analyses and calculations were carried out using GraphPad Prism 5 software. Statistical significance was set at *p<0.05.

### Ethics

Brain tissue samples were obtained from the Institute of Neuropathology Brain Bank (HUB-ICO-IDIBELL Biobank) and the Biobank of Hospital Clinic-IDIBAPS, following pertinent guidelines of the Spanish legislation and the local ethics committee. The present study was conducted according to the revised Declaration of Helsinki and Good Clinical Practice guidelines and was approved by the local ethics committees (University of Göttingen -No. 9/6/08, 19/11/09 and 18/8/15). Informed written consent was given by all study participants or their legal representative. All participants were adults, and samples were anonymized.

For animal investigation, principles of laboratory animal care (NIH publication No. 86–23, revised 1985) were followed. All animal experiments were performed in compliance with the French, national guidelines, in accordance with the European Community Council Directive 86/609/EEC. The protocols comply with the Animal Research: Reporting In Vivo Experiments (ARRIVE) guidelines. The experimental protocol was approved by the INRA Toulouse/ENVT ethics committee (Permit number: 310955547).

## Supporting information

S1 Fig MA plots from small RNA-Seq in the FC and CB of control, sCJD MM1 and VV2 cases. Plots represent the log2 fold change over the mean expression of mean normalized counts for each group comparison.
(TIF)

S2 Fig Distribution of the different types of isomiRs in control and sCJD. IsomiR distribution in control individuals (red) and MM1 or VV2 samples (blue), in the FC (A-B) and the CB (C-D). The size of the dots shows the relative abundance of the different types of isomiRs. The Y-axis shows the fraction of unique sequences affecting specific nucleotides along the miRNAs, indicated in the X-axis. In the X-axis the nucleotide changes at diverse positions defining the isomiR are indicated with respect the reference miRNA (the miRbase sequence). For the trimming variants positions with a minus refer positions upstream of the reference miRNA.
(TIF)

S3 Fig Expression of GW182 in sCJD brain tissue. (A) Gene expression levels of GW182 (n = 10) in the FC and CB of controls, sCJD MM1 and VV2 cases by RT-qPCR. Results were normalized to housekeeping genes GAPDH (figure) and GUSB with similar results. Housekeeping levels remained unaltered between groups. (B) Protein levels of GW182 in the FC of controls, sCJD MM1 and VV2 cases, by western blot analysis. Three representative cases per diagnostic group and brain region are shown in the western blot. Quantifications derived from densitometry analysis were performed in 15 cases per diagnostic group (n = 15/group). β-actin was used as a loading control. Densitometries of the western blots (n = 15 cases/group) are shown. Normalization was performed relative to controls. Error bars indicate SD. In all cases, statistical significance (compared to controls) was set at *p<0.05. (C) Representative fluorescence photomicrographs of GW182 immunoreactivity in the FC of control, sCJD MM1 and sCJD VV2 cases. Phase contrast and merge images are shown. Scale bar = 25μm.
(TIF)

S4 Fig Analysis of gel filtration chromatography fractions from sCJD cases. (A) Coomassie-blue staining of the chromatographic gel filtration fractions obtained from the FC of a sCJD brain homogenate. A representative image of three independent experiments is shown. Molecular weight and fraction numbers are indicated (B) Western blot analysis of the different gel filtration fractions using the Prion protein SAF70 antibody. A representative control and sCJD case is shown. (C) RT-QuIC analysis of the input and chromatographic gel filtration fractions 2 and 7 obtained from the FC of a control and of sCJD brain homogenate. Positive signal was only detected in sCJD fractions. Higher signal, as detected by shorter lag phase was detected in the input, fraction 2 and fraction 7, respectively. A representative image of three independent experiments is shown. (D) Dot-blot analysis developed against the oligomer antibody 11A of the input and chromatographic gel filtration fractions 2 and 7 obtained from the FC of a control and sCJD brain homogenate. Positive signal was only detectable in sCJD fractions. A representative image of three independent experiments is shown.
(TIF)

S5 Fig Subcellular localization of Ago-2 in sCJD brain tissue. **(**A) Representative fluorescence photomicrographs of Ago-2 immunoreactivity (11A9 antibody) in the FC of control, sCJD MM1 and sCJD VV2 cases. Phase contrast and merge images are shown. Scale bar = 25μm. Representative fluorescence photomicrographs of Ago-2 (11A9 antibody, green channel) and Iba-1 (red channel) immunoreactivity in the FC of control and sCJD MM1 cases. Arrowheads indicate absence of Ago-2 expression in microglial cells in control cases. In contrast, in sCJD cases some microglial cells are double stained with Ago-2 antibody. Merge images are shown. Scale bar = 25μm (C).
(TIF)

S6 Fig Expression of Exportin 5 in sCJD brain tissue. (A) Gene expression levels of Exportin 5 (n = 10) in the FC and CB of controls, sCJD MM1 and VV2 cases by RT-qPCR. Results were normalized to housekeeping genes GAPDH (figure) and GUSB with similar results. Housekeeping levels remained unaltered between groups. (B) Protein levels of Exportin 5 in the FC of controls, sCJD MM1 and VV2 cases, by western blot analysis. Two representative cases per diagnostic group and brain region are shown in the western blot. Quantifications derived from densitometry analysis were performed in 15 cases per diagnostic group (n = 15/group). β-actin was used as a loading control. Densitometries of the western blot (n = 15 cases/group) are shown. Normalization was performed relative to controls. Error bars indicate SD. In all cases, statistical significance (compared to controls) was set at *p<0.05.
(TIF)

S7 Fig Synaptic loss in the sCJD MM1 mouse model tg340-PRNP129MM. Western blot analysis of PSD-95 and synaptophysin in the cortex and CB of control and sCJD MM1 inoculated mice at different disease stages. GAPDH was used as a loading control.
(TIF)

S8 Fig Temporal-dependent expression of validated miRNAs in the sCJD MM1 mouse model tg340-PRNP129MM. Representation of the miRNAs with regulated expression levels in the cortex (A) and CB (B) of the tg340 mice by RT-qPCR analysis at any of the different stages of the disease. Data is represented as the fold change between sCJD MM1 and control inoculated animals and each colored line represents a miRNA.
(TIF)

S9 Fig Selective cell death induction by sCJD miRNAs transfection in cell cultures. (A) miRNAs from Control, sCJD MM1 and sCJD VV2 cases (FC region) were highly purified using commercial RNA extraction kits followed by in-gel purification and transfection into SH-SY5Y RA-differentiated cells and glioblastoma H4 cells. RNA was extracted from the cells and selected sCJD-related miRNAs were quantified by qPCR. NT: non-transfected. Statistical differences of comparison with non-transfected cells are indicated; p<0.05. (B) Cell toxicity assay, using Iodide Propidium staining (PI) on H4 and SY-SY5Y cells at 48h post-transfection with highly purified miRNAs derived from Control, sCJD MM1 and sCJD VV2 cases (FC region). (C) Cell viability, using WST-1 on SY-SY5Y cells at 48h post-transfection transfected with highly purified miRNAs derived from Control, sCJD MM1 and sCJD VV2 cases (FC region). Statistical differences are referred to controls. Statistical significance was set at *p<0.05.
(TIF)

S1 Table Excel file including the total RNA-sequence reads in each case (online). (XLSX)

S2 Table Excel file including the total number of reads mapping onto miRNAs. miRNAs with at least 2 counts in a given sample (online).
(XLSX)

S3 Table Excel file including regulated miRNAs detected in this study (online). (XLSX)

S4 Table Excel file displaying isomiRs detected in this study. Base mean > or equal to 10 (online).
(XLSX)

S5 Table Table providing information on the functional enrichment analysis of miRNAs identified based on small RNA-Seq in this study. (TIF)

S6 Table Top transcription factor binding sites enriched for promoter regions of target downregulated (A) and upregulated (B) miRNAs in the FC of sCJD MM1 cases.
(TIF)

S7 Table List of Taqman probes, miRCURY LNA primers and antibodies used in this study. (TIF)

S8 Table Brain samples used in the present study. Diagnostic, sCJD subtype, brain region, age, sex and the type of experiment for which each case was used (RNA-seq, miRNA qPCR, mRNA qPCR and western blot) is indicated.
(TIF)

S9 Table Demographics and biomarker profiling for the CSF samples analyzed in this study. NA = Non-analyzed, + = positive according to sCJD cut-off, − = negative according to sCJD cut-off. M = male, F = female.
(TIF)
