# Supplementary figures and images for: Regional and subtype-dependent miRNA signatures in sporadic Creutzfeldt-Jakob disease are accompanied by alterations in miRNA silencing machinery and biogenesis
Source: PLoS Pathog. 2018 Jan 22;14(1):e1006802. doi: 10.1371/journal.ppat.1006802 (PMC5794191; doi:10.1371/journal.ppat.1006802)

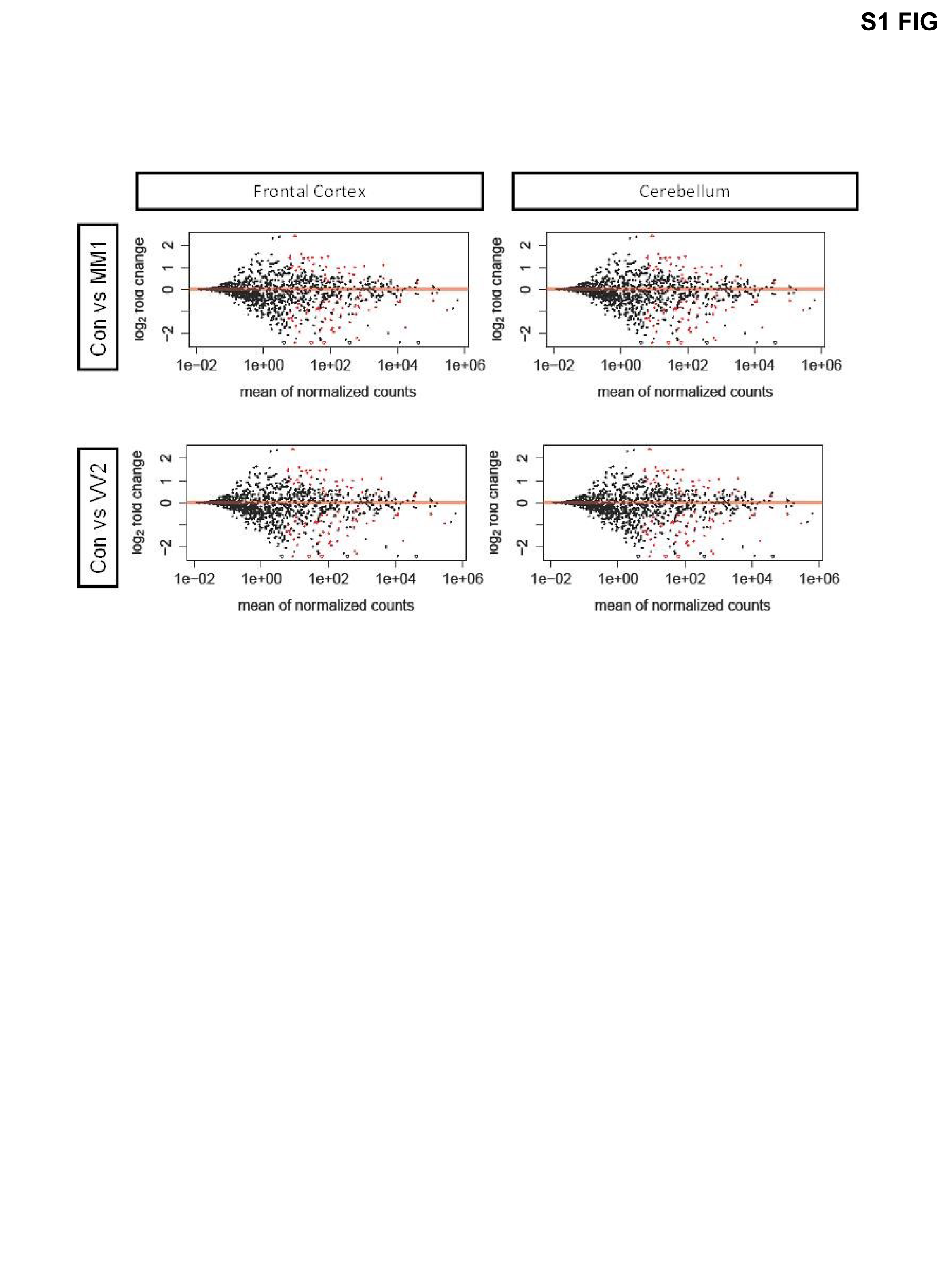

Supplement: S1 Fig — Plots represent the log2 fold change over the mean expression of mean normalized counts for each group comparison. (TIF) [file ppat.1006802.s001.tif]

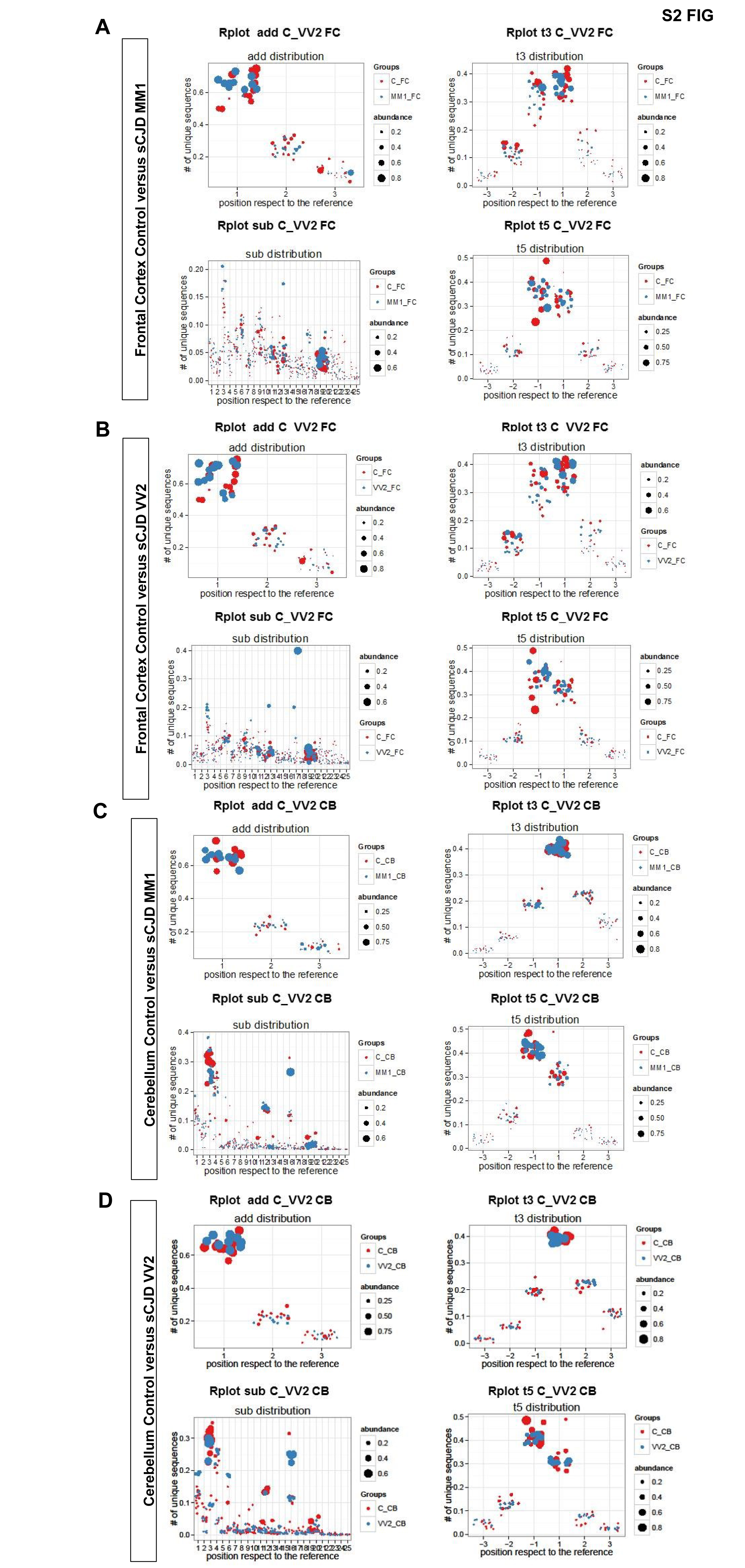

Supplement: S2 Fig — IsomiR distribution in control individuals (red) and MM1 or VV2 samples (blue), in the FC (A-B) and the CB (C-D). The size of the dots shows the relative abundance of the different types of isomiRs. The Y-axis shows the fraction of unique sequences affecting specific nucleotides along the miRNAs, indicated in the X-axis. In the X-axis the nucleotide changes at diverse positions defining the isomiR are indicated with respect the reference miRNA (the miRbase sequence). For the trimming variants positions with a minus refer positions upstream of the reference miRNA. (TIF) [file ppat.1006802.s002.tif]

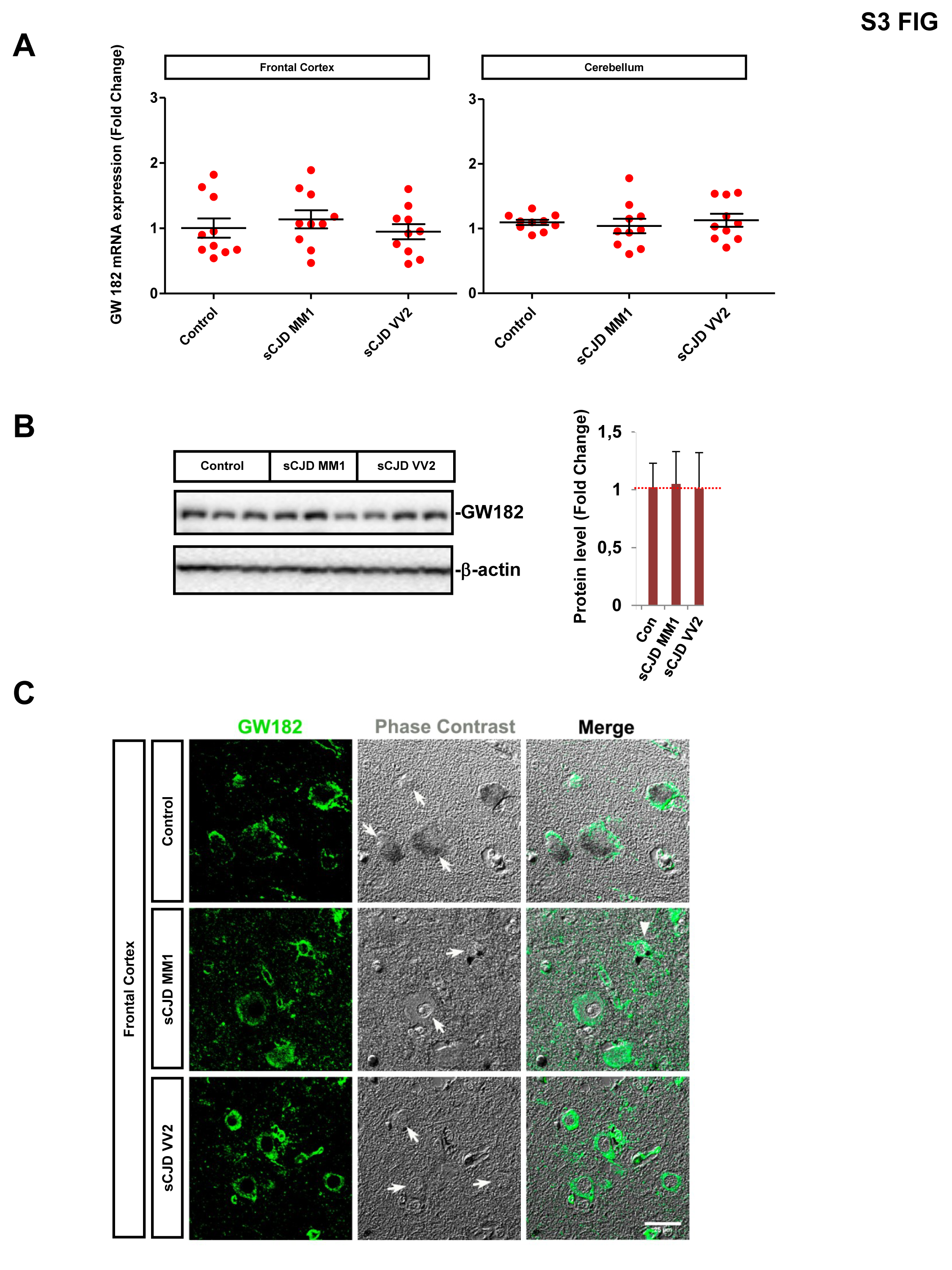

Supplement: S3 Fig — (A) Gene expression levels of GW182 (n = 10) in the FC and CB of controls, sCJD MM1 and VV2 cases by RT-qPCR. Results were normalized to housekeeping genes GAPDH (figure) and GUSB with similar results. Housekeeping levels remained unaltered between groups. (B) Protein levels of GW182 in the FC of controls, sCJD MM1 and VV2 cases, by western blot analysis. Three representative cases per diagnostic group and brain region are shown in the western blot. Quantifications derived from densitometry analysis were performed in 15 cases per diagnostic group (n = 15/group). β-actin was used as a loading control. Densitometries of the western blots (n = 15 cases/group) are shown. Normalization was performed relative to controls. Error bars indicate SD. In all cases, statistical significance (compared to controls) was set at *p<0.05. (C) Representative fluorescence photomicrographs of GW182 immunoreactivity in the FC of control, sCJD MM1 and sCJD VV2 cases. Phase contrast and merge images are shown. Scale bar = 25μm. (TIF) [file ppat.1006802.s003.tif]

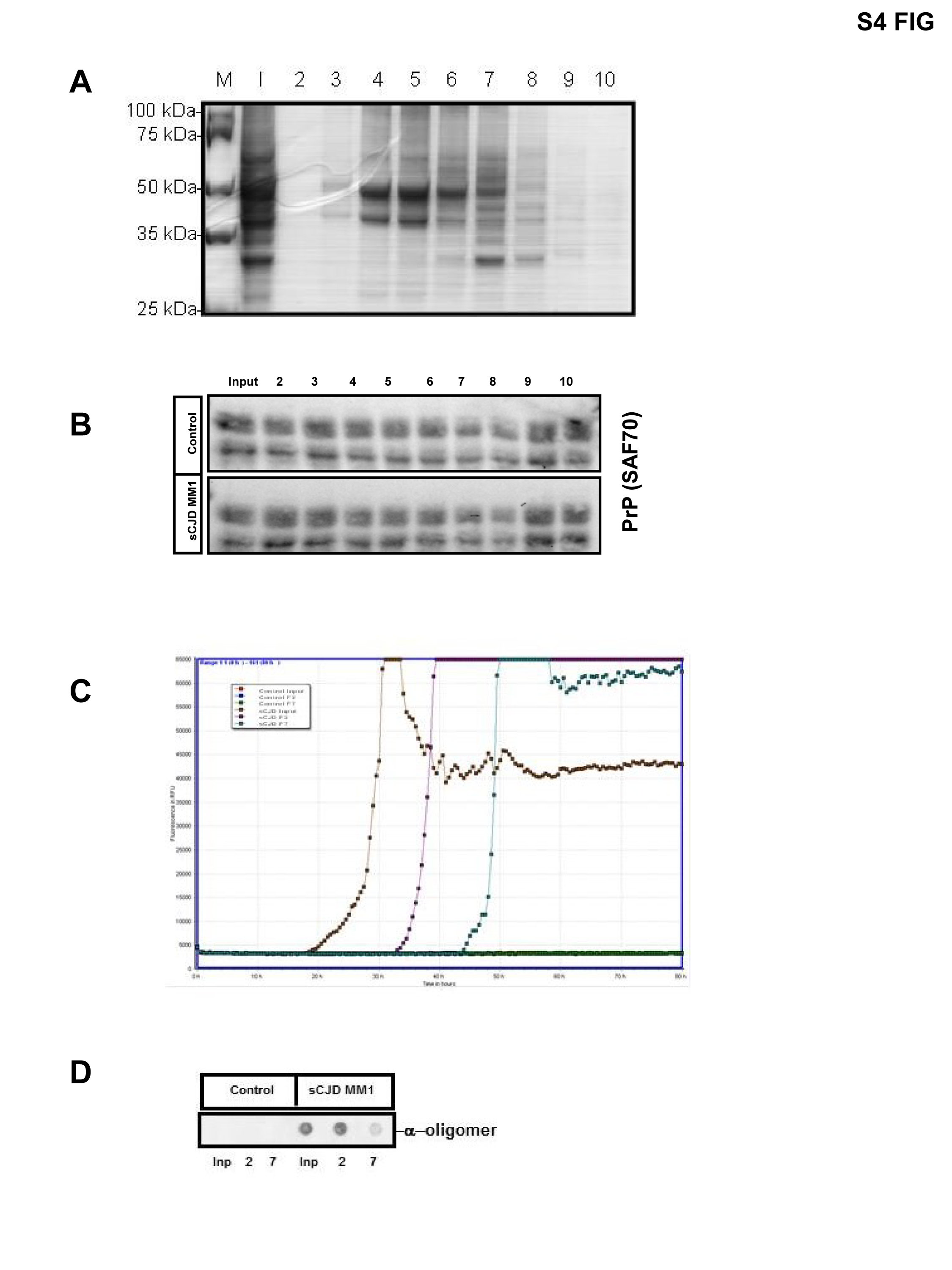

Supplement: S4 Fig — (A) Coomassie-blue staining of the chromatographic gel filtration fractions obtained from the FC of a sCJD brain homogenate. A representative image of three independent experiments is shown. Molecular weight and fraction numbers are indicated (B) Western blot analysis of the different gel filtration fractions using the Prion protein SAF70 antibody. A representative control and sCJD case is shown. (C) RT-QuIC analysis of the input and chromatographic gel filtration fractions 2 and 7 obtained from the FC of a control and of sCJD brain homogenate. Positive signal was only detected in sCJD fractions. Higher signal, as detected by shorter lag phase was detected in the input, fraction 2 and fraction 7, respectively. A representative image of three independent experiments is shown. (D) Dot-blot analysis developed against the oligomer antibody 11A of the input and chromatographic gel filtration fractions 2 and 7 obtained from the FC of a control and sCJD brain homogenate. Positive signal was only detectable in sCJD fractions. A representative image of three independent experiments is shown. (TIF) [file ppat.1006802.s004.tif]

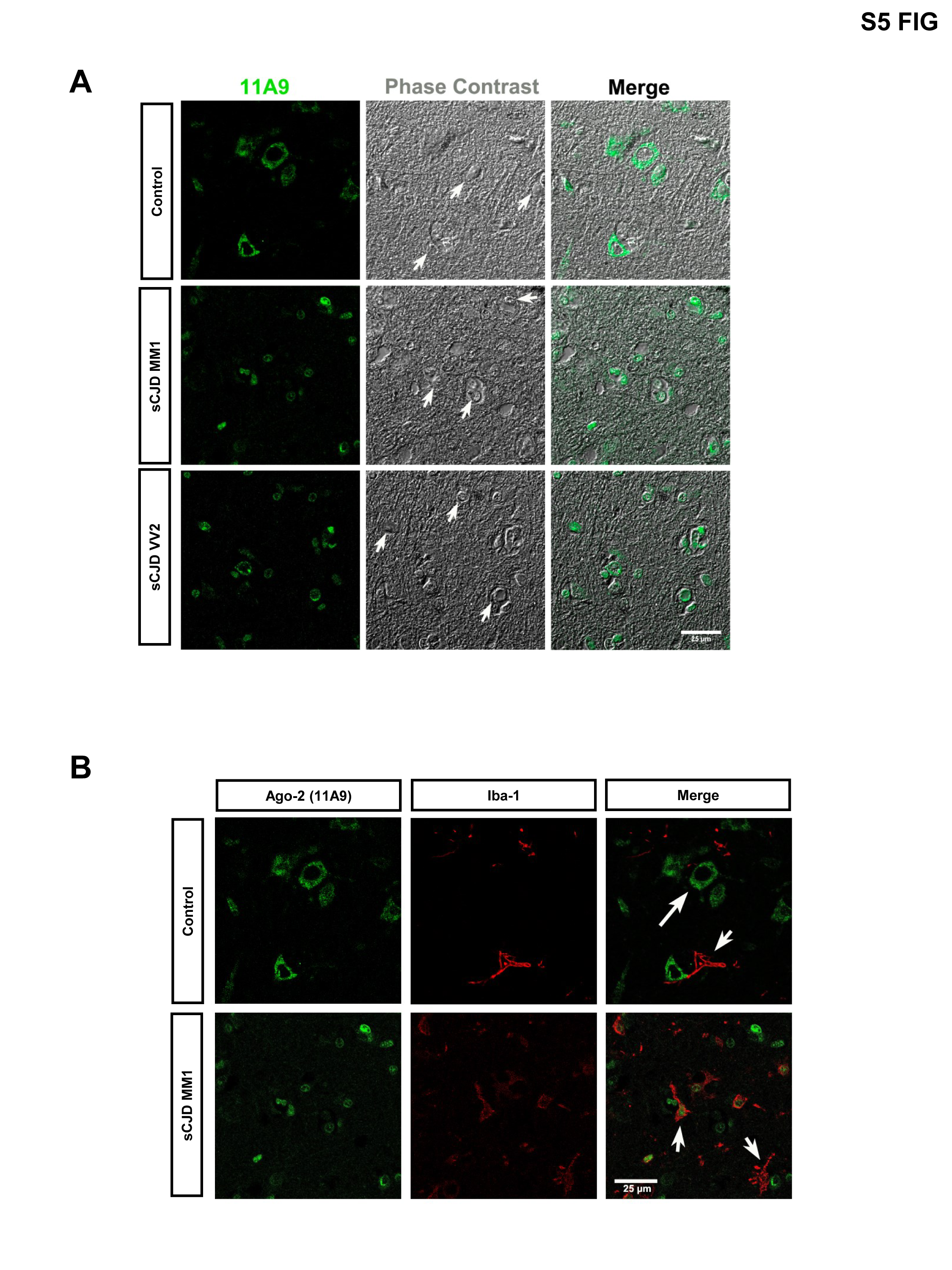

Supplement: S5 Fig — (A) Representative fluorescence photomicrographs of Ago-2 immunoreactivity (11A9 antibody) in the FC of control, sCJD MM1 and sCJD VV2 cases. Phase contrast and merge images are shown. Scale bar = 25μm. Representative fluorescence photomicrographs of Ago-2 (11A9 antibody, green channel) and Iba-1 (red channel) immunoreactivity in the FC of control and sCJD MM1 cases. Arrowheads indicate absence of Ago-2 expression in microglial cells in control cases. In contrast, in sCJD cases some microglial cells are double stained with Ago-2 antibody. Merge images are shown. Scale bar = 25μm (C). (TIF) [file ppat.1006802.s005.tif]

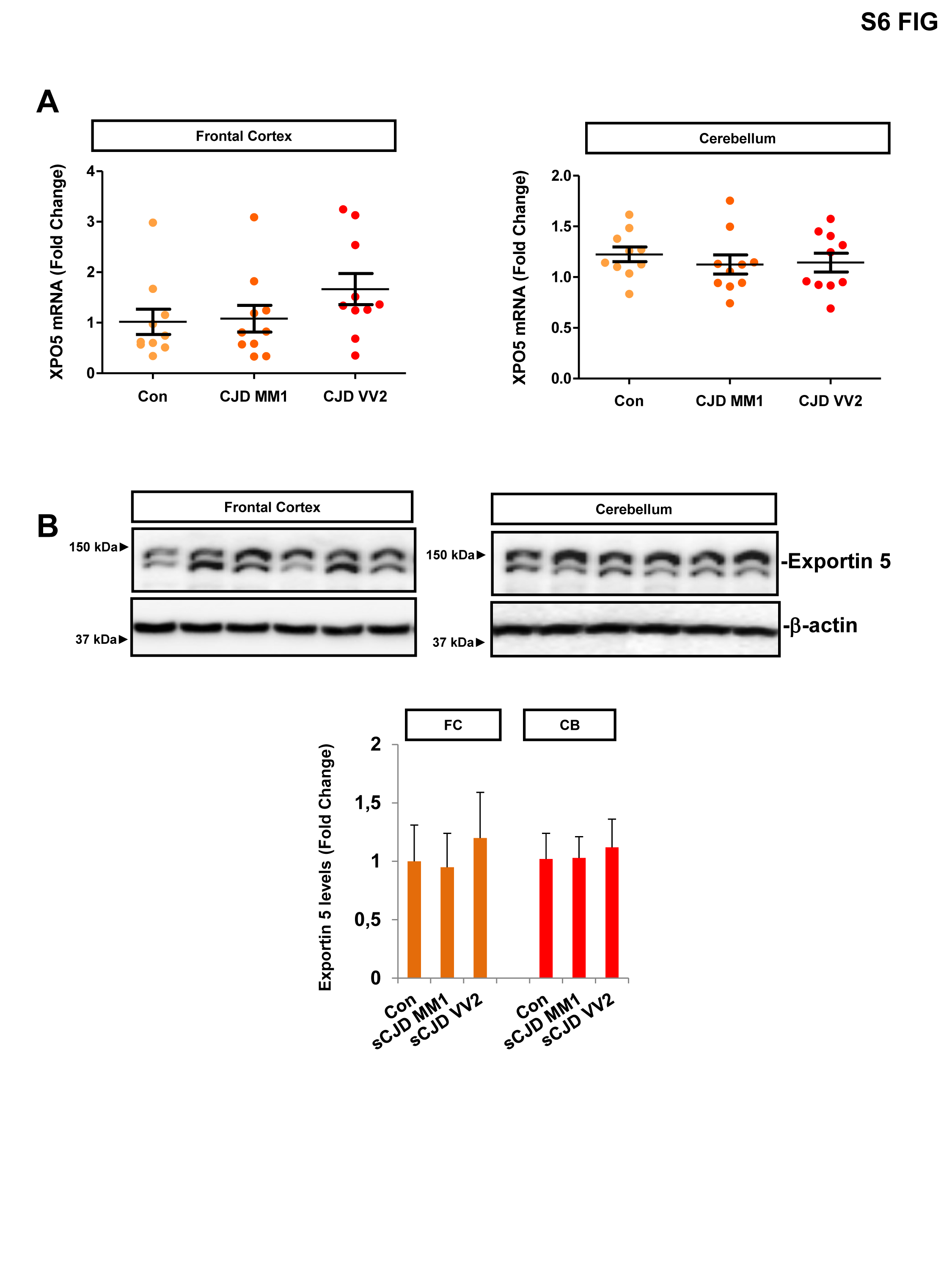

Supplement: S6 Fig — (A) Gene expression levels of Exportin 5 (n = 10) in the FC and CB of controls, sCJD MM1 and VV2 cases by RT-qPCR. Results were normalized to housekeeping genes GAPDH (figure) and GUSB with similar results. Housekeeping levels remained unaltered between groups. (B) Protein levels of Exportin 5 in the FC of controls, sCJD MM1 and VV2 cases, by western blot analysis. Two representative cases per diagnostic group and brain region are shown in the western blot. Quantifications derived from densitometry analysis were performed in 15 cases per diagnostic group (n = 15/group). β-actin was used as a loading control. Densitometries of the western blot (n = 15 cases/group) are shown. Normalization was performed relative to controls. Error bars indicate SD. In all cases, statistical significance (compared to controls) was set at *p<0.05. (TIF) [file ppat.1006802.s006.tif]

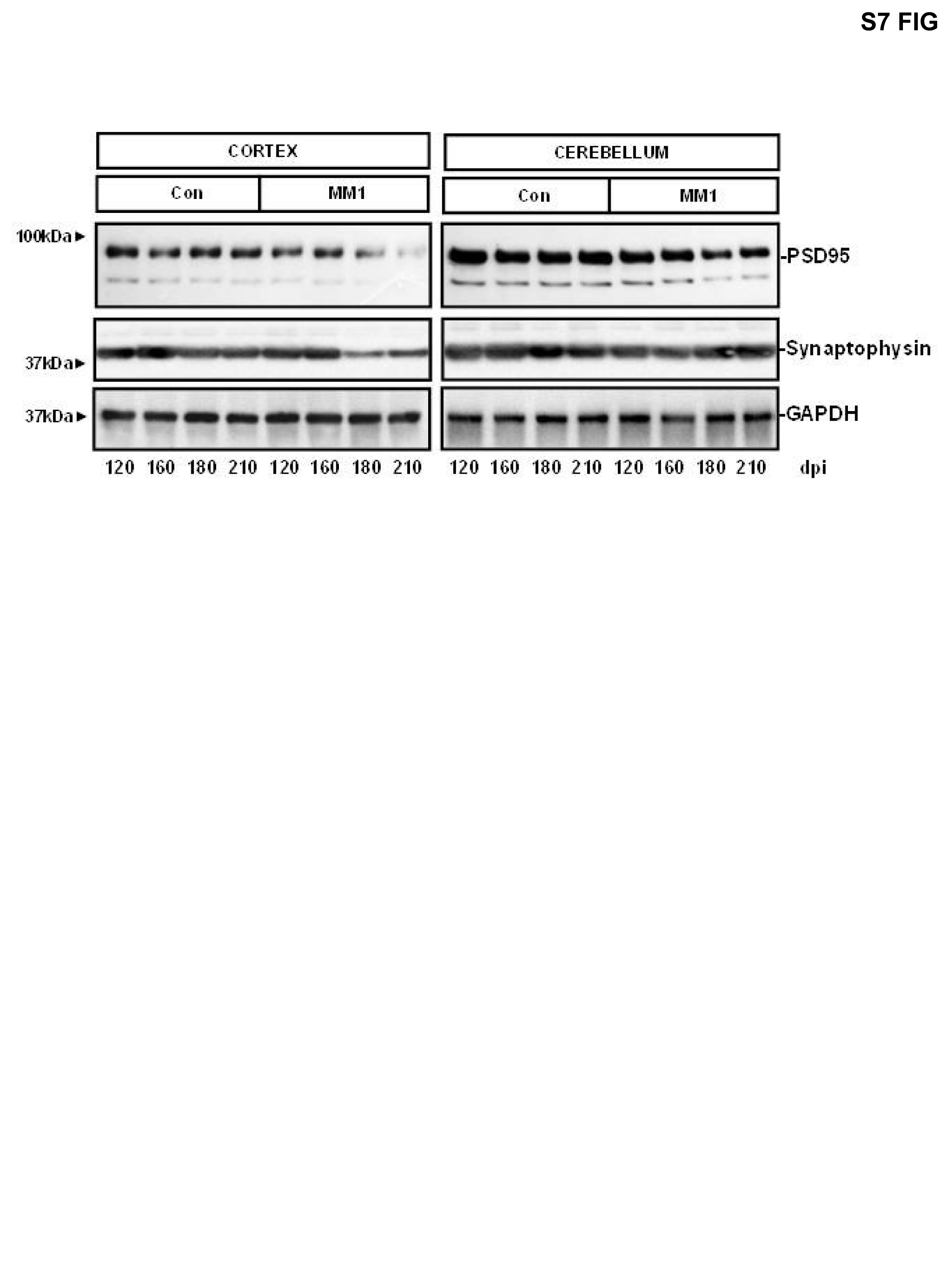

Supplement: S7 Fig — Western blot analysis of PSD-95 and synaptophysin in the cortex and CB of control and sCJD MM1 inoculated mice at different disease stages. GAPDH was used as a loading control. (TIF) [file ppat.1006802.s007.tif]

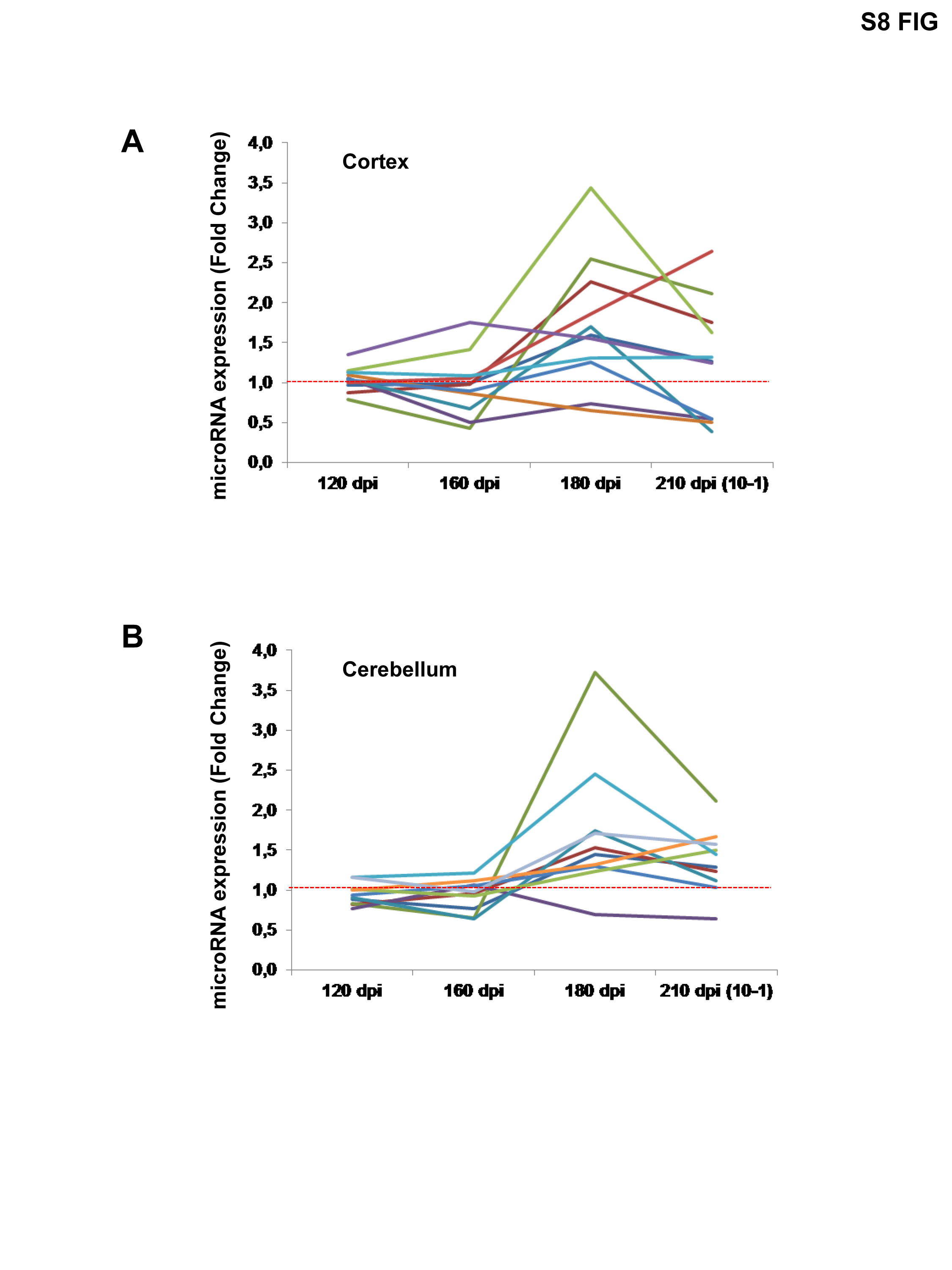

Supplement: S8 Fig — Representation of the miRNAs with regulated expression levels in the cortex (A) and CB (B) of the tg340 mice by RT-qPCR analysis at any of the different stages of the disease. Data is represented as the fold change between sCJD MM1 and control inoculated animals and each colored line represents a miRNA. (TIF) [file ppat.1006802.s008.tif]

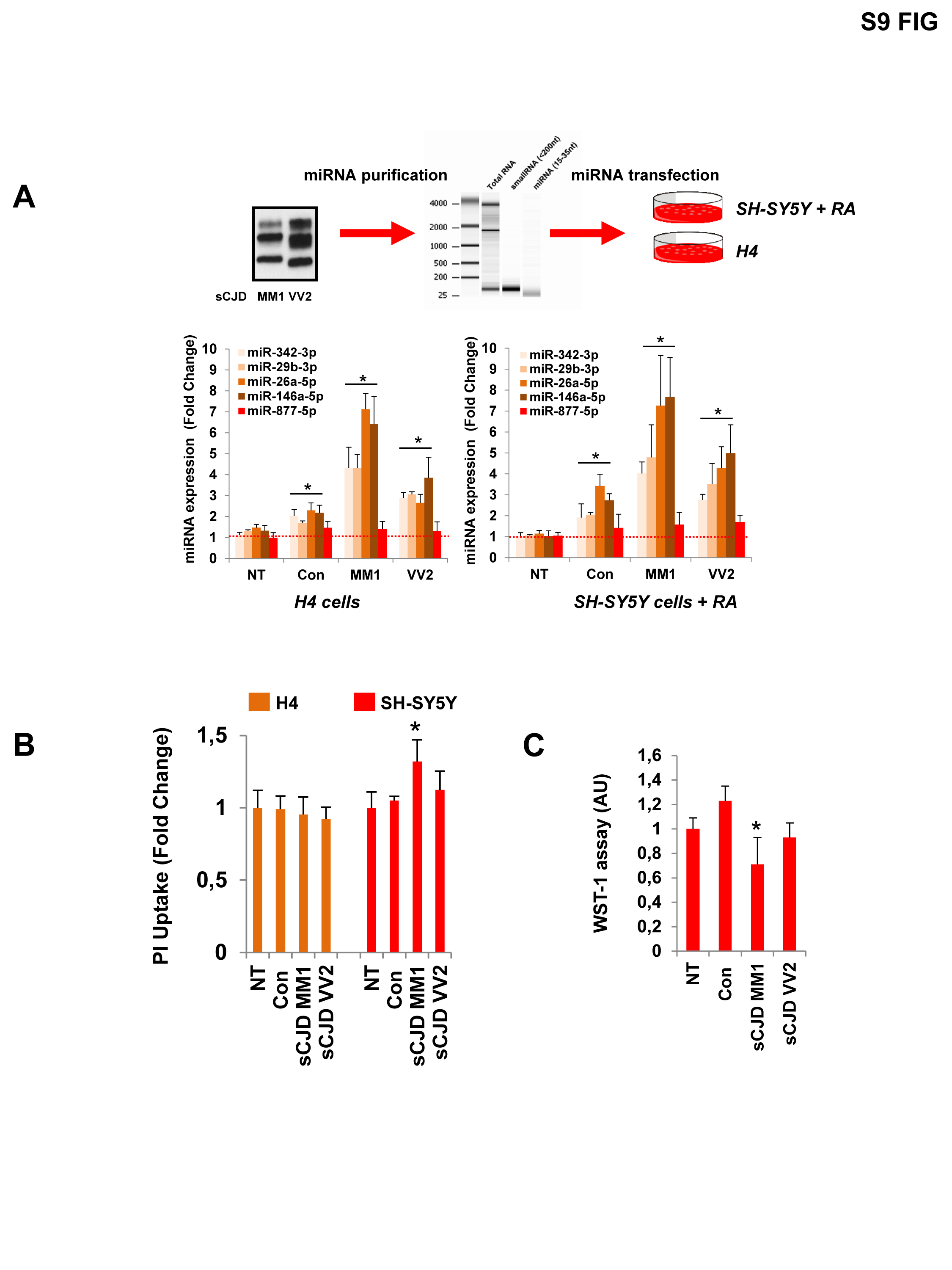

Supplement: S9 Fig — (A) miRNAs from Control, sCJD MM1 and sCJD VV2 cases (FC region) were highly purified using commercial RNA extraction kits followed by in-gel purification and transfection into SH-SY5Y RA-differentiated cells and glioblastoma H4 cells. RNA was extracted from the cells and selected sCJD-related miRNAs were quantified by qPCR. NT: non-transfected. Statistical differences of comparison with non-transfected cells are indicated; p<0.05. (B) Cell toxicity assay, using Iodide Propidium staining (PI) on H4 and SY-SY5Y cells at 48h post-transfection with highly purified miRNAs derived from Control, sCJD MM1 and sCJD VV2 cases (FC region). (C) Cell viability, using WST-1 on SY-SY5Y cells at 48h post-transfection transfected with highly purified miRNAs derived from Control, sCJD MM1 and sCJD VV2 cases (FC region). Statistical differences are referred to controls. Statistical significance was set at *p<0.05. (TIF) [file ppat.1006802.s009.tif]

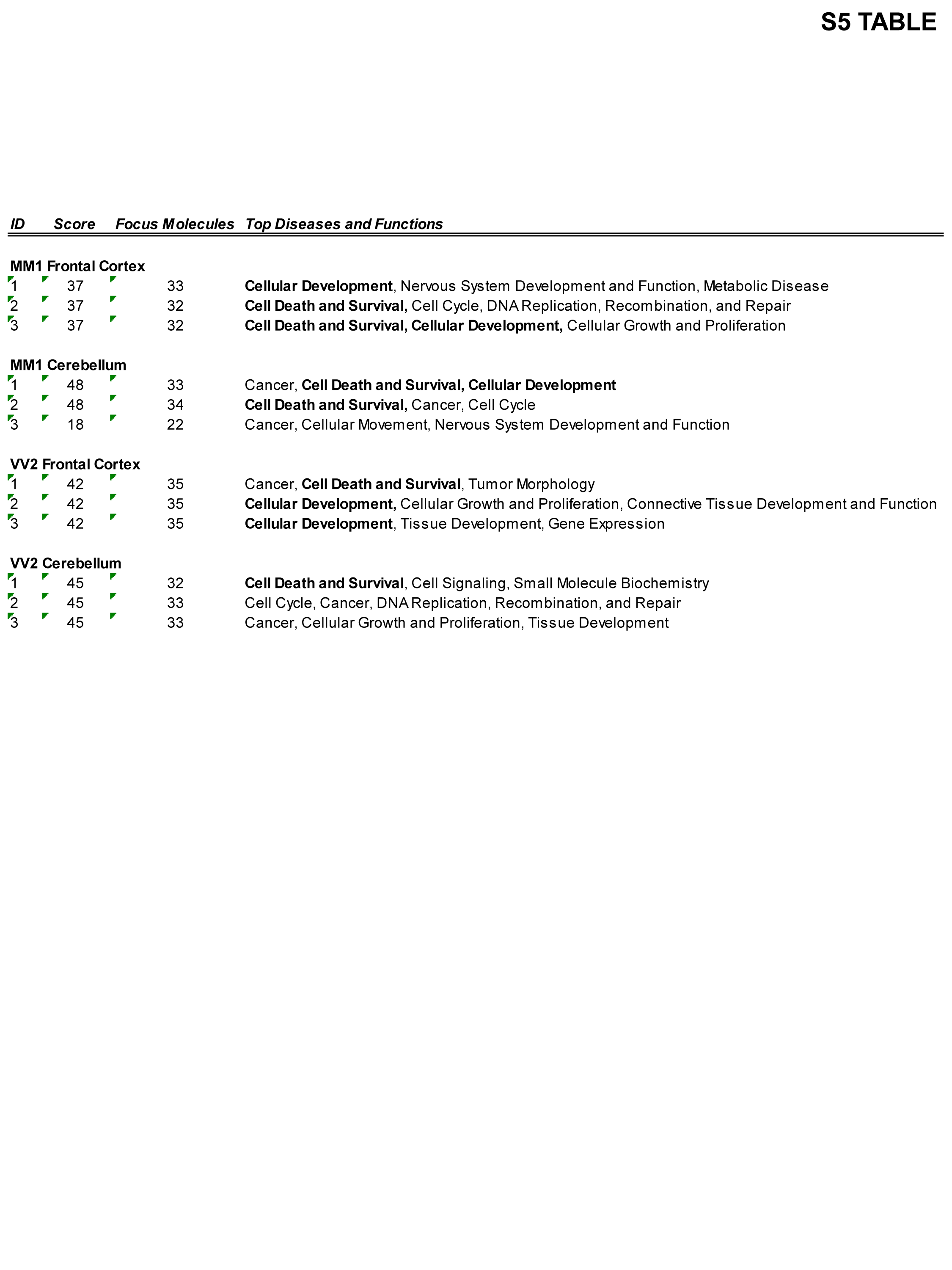

Supplement: S5 Table — (TIF) [file ppat.1006802.s014.tif]

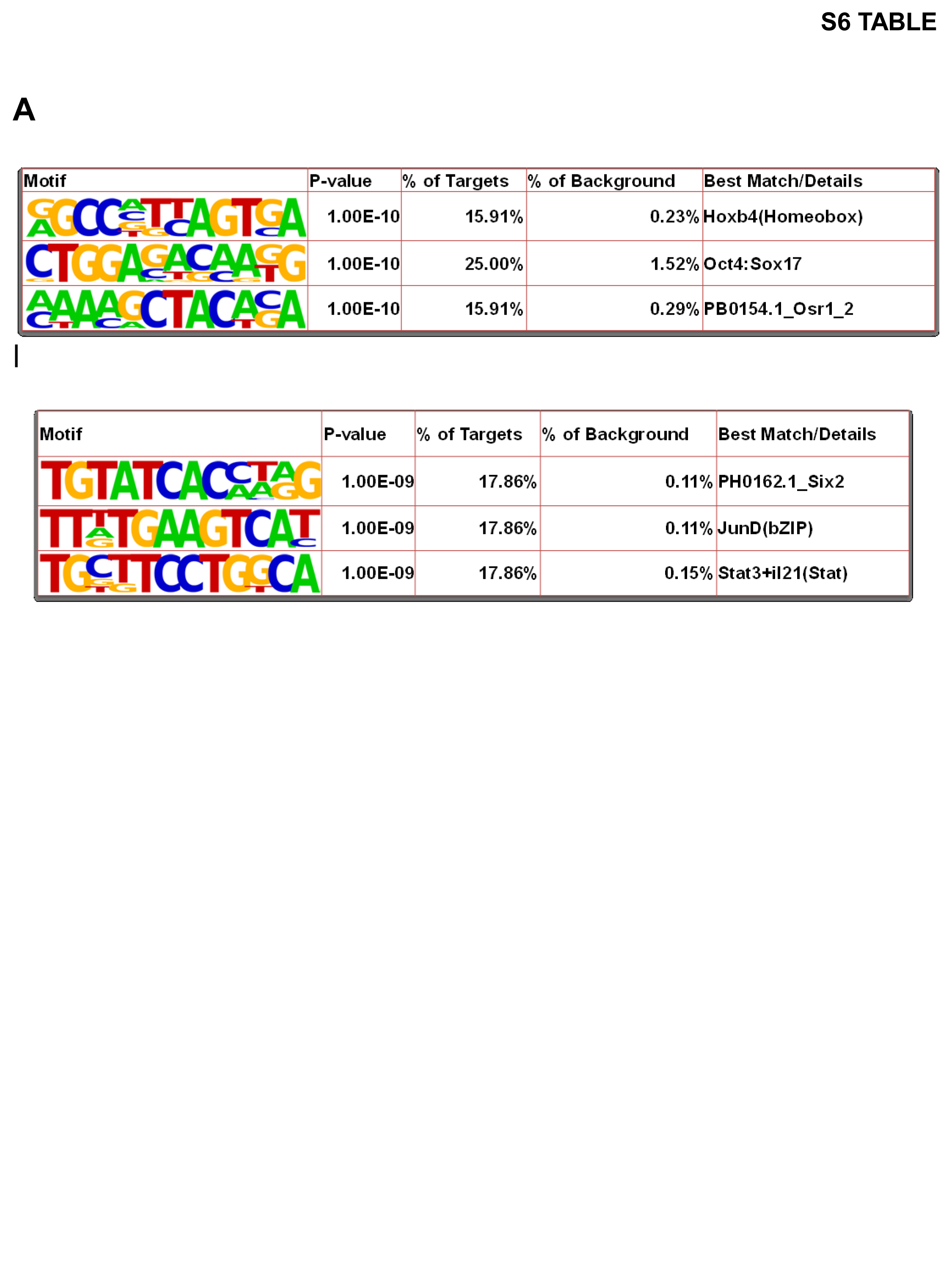

Supplement: S6 Table — Top transcription factor binding sites enriched for promoter regions of target downregulated (A) and upregulated (B) miRNAs in the FC of sCJD MM1 cases. (TIF) [file ppat.1006802.s015.tif]

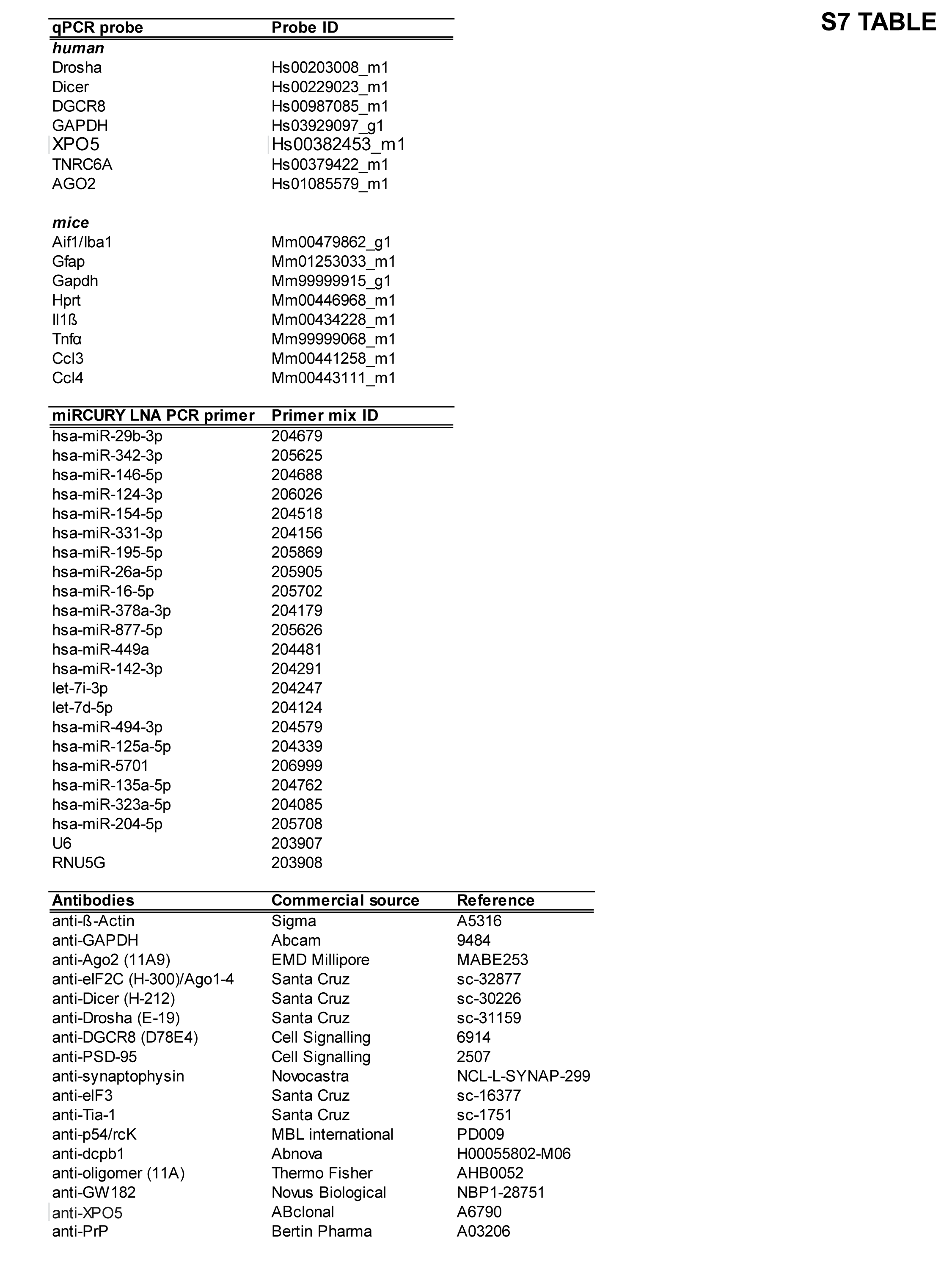

Supplement: S7 Table — (TIF) [file ppat.1006802.s016.tif]

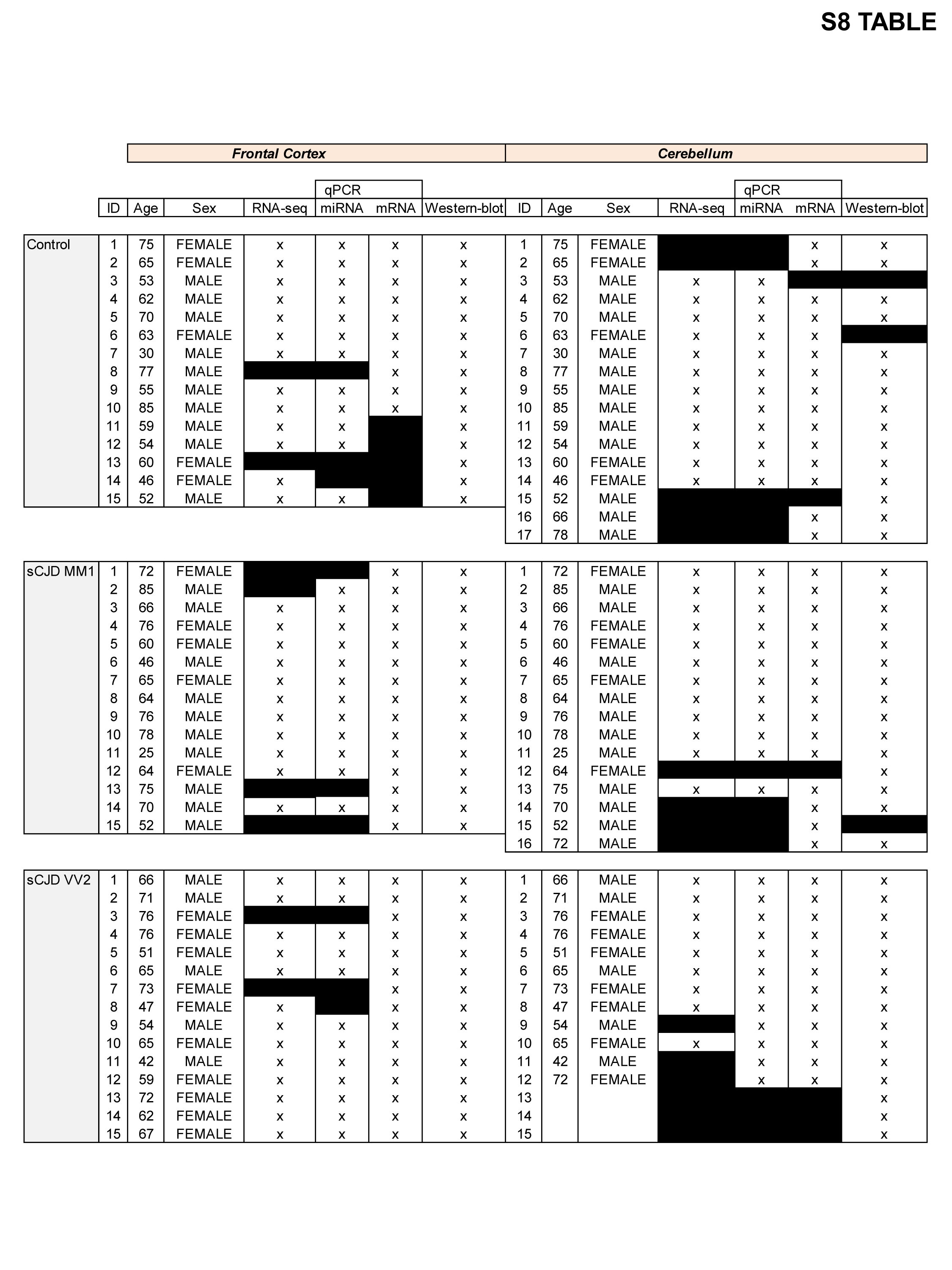

Supplement: S8 Table — Diagnostic, sCJD subtype, brain region, age, sex and the type of experiment for which each case was used (RNA-seq, miRNA qPCR, mRNA qPCR and western blot) is indicated. (TIF) [file ppat.1006802.s017.tif]

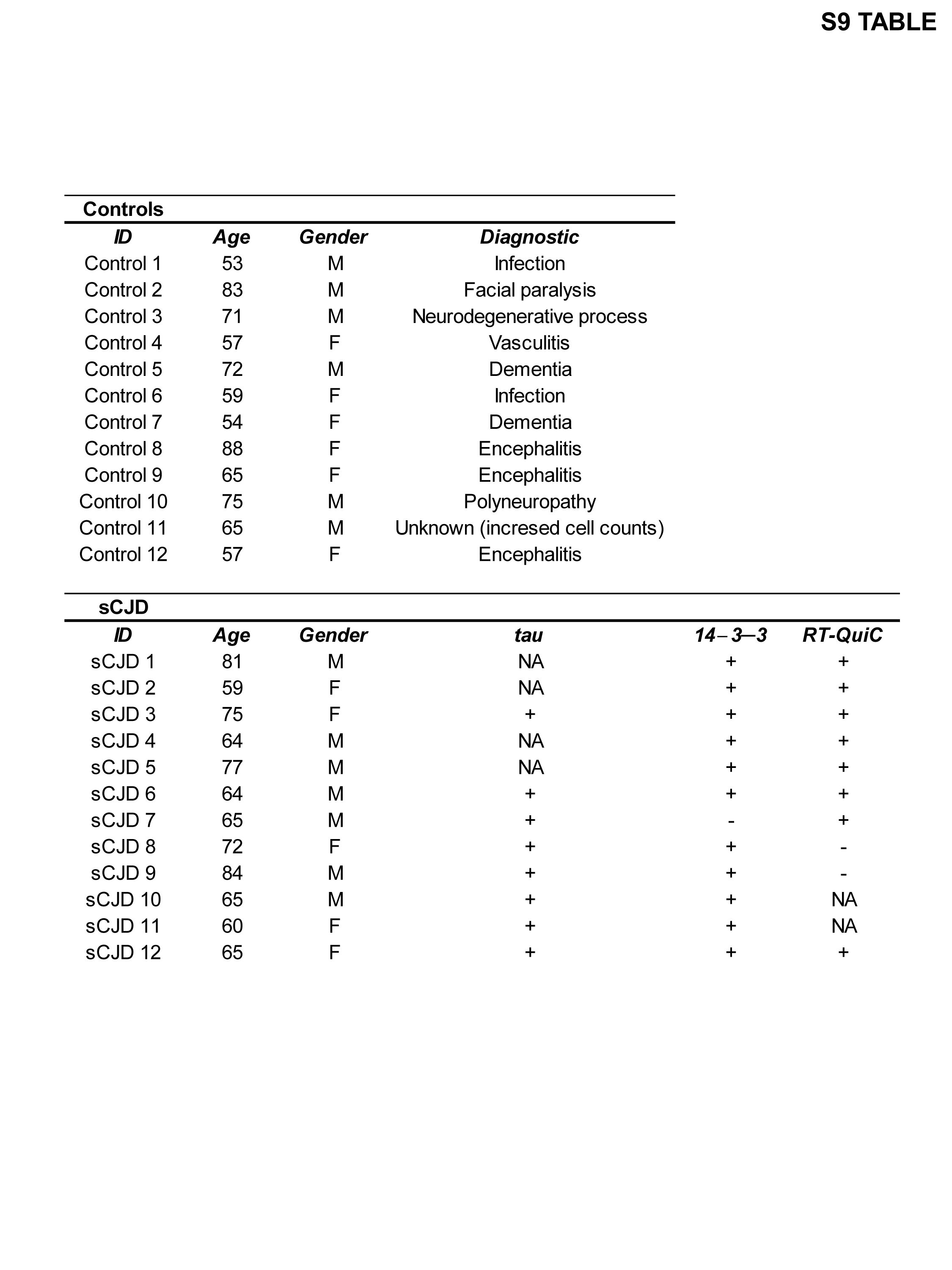

Supplement: S9 Table — NA = Non-analyzed, + = positive according to sCJD cut-off, − = negative according to sCJD cut-off. M = male, F = female. (TIF) [file ppat.1006802.s018.tif]
